# PRIME: An evaluation framework for protein representation inference and generalization in viral mutation space

**DOI:** 10.1186/s12864-026-12976-5

**Published:** 2026-05-30

**Authors:** Kaetlyn Gibson, Po-E Li, Valerie Li, Martha Dix, Li-Wei Hung, George Widgery Stelle, Michal Babinski, Patrick Chain, Bin Hu

**Affiliations:** 1 Genomics and Bioanalytics Group, Bioscience Division, Los Alamos National Laboratory, Los Alamos, NM 87545 USA; 2 Biochemistry and Biotechnology Group, Bioscience Division, Los Alamos National Laboratory, Los Alamos, NM 87545 USA; 3 Applied Computer Science Group, Computer, Computational and Statistical Sciences Division, Los Alamos National Laboratory, Los Alamos, NM 87545 USA

**Keywords:** Protein language models, Biosurveillance, Phenotype prediction, Gomology leakage, Position-stratified validation, Host tropism prediction, Model fine-tuning, Machine learning efficiency

## Abstract

**Background:**

Protein language models (PLMs) have revolutionized protein fitness prediction, yet their application to rapidly evolving viral pathogens is often confounded by extreme sequence homology. This homology leads to “data leakage” in standard random validation splits, yielding inflated performance metrics that fail to translate into real-world biosurveillance utility.

**Results:**

We present Protein Representation Inference for Mutation Evaluation (PRIME), a framework that integrates domain-specific fine-tuning with a rigorous position-stratified validation protocol to evaluate viral threats. Using a dataset of 347,432 SARS-CoV-2 receptor binding domain (RBD) sequences, we demonstrate that while random training data split yields deceptive R^2^ values (> 0.90), they fail to generalize to novel mutational sites. By benchmarking models up to 650 M parameters, we show that domain-specific fine-tuning of the ESM-C 600 M model with correctly stratified data provides an initial demonstration of predictive signal for binding affinity and expression at unseen mutational sites of binding affinity and expression on unseen sites (R^2^ ~0.23), a significant advancement over base foundation models which exhibit no predictive power (R^2^ <0). PRIME’s embedding-based clustering identified 3.03% of bat coronavirus sequences as candidates for further experimental prioritization based on their functional similarity to human-infective strains in embedding space, offering a perspective complementary to traditional phylogenetic methods.

**Conclusion:**

PRIME establishes a new benchmark for the application of PLMs in pathogen surveillance. Our findings demonstrate that state-of-the-art models and fine-tuning, when paired with stratified validation, provide biologically meaningful insights into pathogen evolution and zoonotic risk.

**Supplementary Information:**

The online version contains supplementary material available at 10.1186/s12864-026-12976-5.

## Background

Pathogen evolution continuously generates variants with altered phenotypic properties that fundamentally shift transmission dynamics [1–5], immune interactions [6–9], and host range [10–12]. Current computational biosurveillance strategies, while effective for phylogenetic tracking, face significant limitations in predicting how specific genetic changes translate into the phenotypic consequences that drive outbreak dynamics. Traditional multiple sequence alignment (MSA) and phylogenetic methods [13–16] are inherently reactive; they often fail to capture genotype-to-phenotype relationships between pathogens that share functional properties but are phylogenetically distant [4, 5, 17–19]. Furthermore, these approaches scale poorly [20] when applied to the massive, diverse sequence datasets generated during real-time pandemic monitoring and may introduce artifacts when analyzing hypervariable viral genomes under selective pressure [21]. Recent computational efforts have begun to address this gap through proactive approaches, including machine learning models for forecasting future dominant SARS-CoV-2 lineages [22], anomaly-detection frameworks for flagging emerging variants before they reach epidemiological significance, and generative models that anticipate plausible future viral sequences to expand surveillance coverage beyond observed diversity [23]. While these approaches represent important advances, they have not systematically addressed the evaluation bias introduced by homology leakage, nor have they benchmarked the conditions under which PLM-derived representations genuinely generalize to novel mutational sites.

The receptor-binding domains (RBDs) [24–27] of betacoronavirus spike proteins illustrate these challenges. The SARS-CoV-2 RBD binds the human ACE2 receptor [28] while the related MERS-CoV binds to dipeptidyl peptidase (DPP4) [29, 30], illustrating how discrete sequence variations in homologous domains dictate host specificity. The evolutionary adaptability of the RBD is further underscored by the emergence of the Omicron (B.1.1.529) lineage, where pivotal mutations within the RBD enabled widespread escape by significantly attenuating antibody neutralization efficacy and compromising vaccine-induced immunity [31–34].

Deep mutational scanning (DMS) has emerged as a powerful experimental tool to systematically survey how random mutations across a sequence impacts protein function, providing rich datasets that capture complex, non-linear molecular interactions [35–38]. However, extracting actionable patterns from these high-dimensional, combinatorial datasets requires computational frameworks capable of modeling intricate biophysical constraints.

Protein language models (PLMs) such as the Evolutionary Scale Modeling (ESM) family [39–44], have shown immense promise in capturing sequence-function relationships by learning from millions of protein sequences across the tree of life [45]. Despite their success in general protein modeling and structure prediction, direct application to pathogen-specific sequences often yields limited predictive power. The subtle sequence variations that distinguish viral lineages or confer immune escape represent only a small fraction of the sequence space covered by general foundation models. Moreover, many pathogen sequences are intentionally excluded from the large-scale training sets due to biosecurity concerns, creating a critical resolution gap in legitimate biosurveillance applications.

**Fig. 1:**
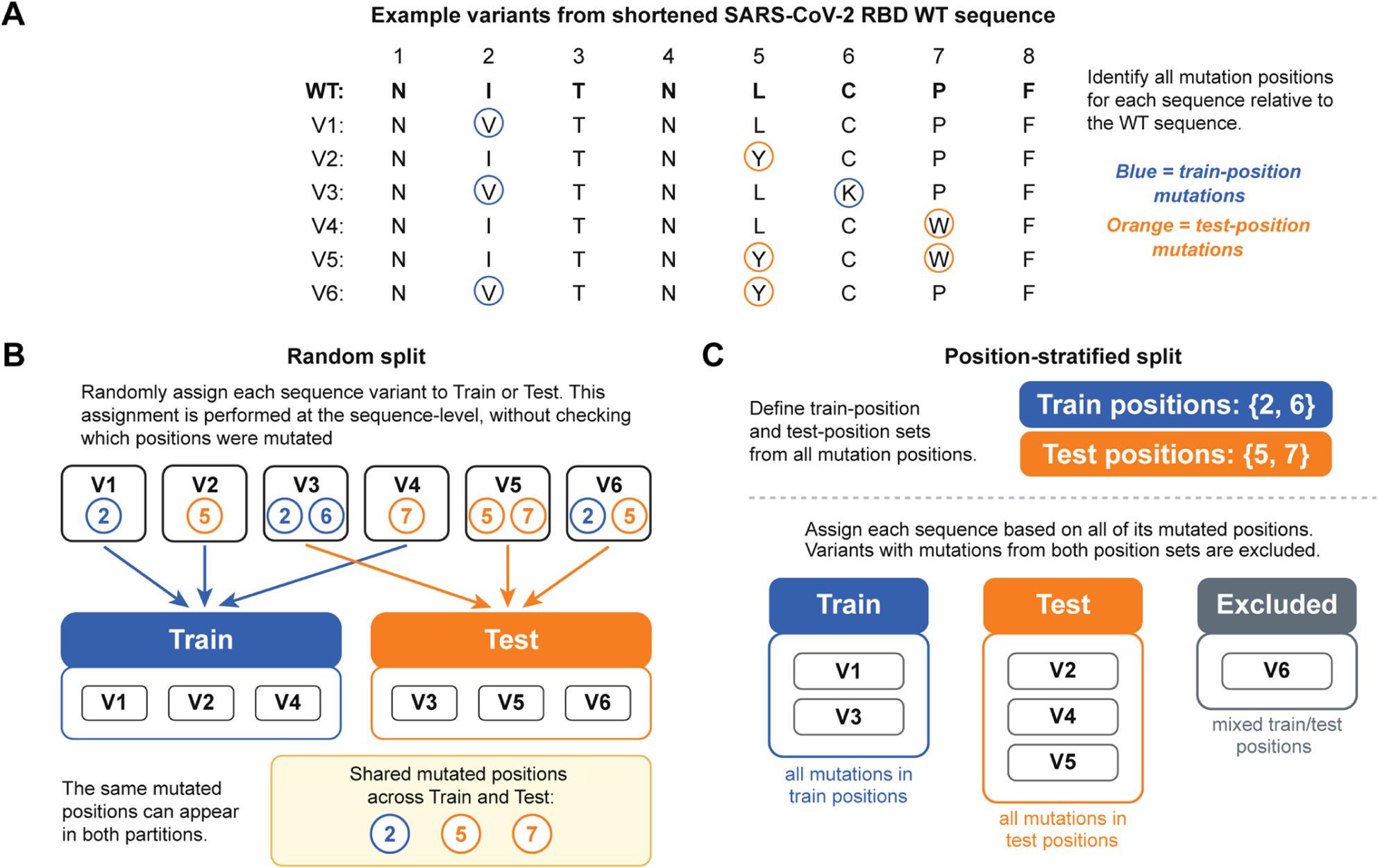
Overview of random and position-stratified splitting strategies for SARS-CoV-2 DMS data. **A** Example sequence variants derived from a shortened wild-type (WT) SARS-CoV-2 RBD sequence. Each variant carries one or more amino acid substitutions (circled) relative to the WT; blue circles indicate positions assigned to the train-position set and orange circles indicate positions assigned to the test-position set. **B** In a random split, variants are assigned to train or test partitions at the sequence level without consideration of mutation positions. Consequently, the same mutated positions appear in both partitions — for example, positions 2, 5, and 7 are shared across train and test — creating positional information leakage that inflates apparent model performance. **C** In a position-stratified split, all mutation positions across the dataset are first identified, then partitioned into disjoint train-position and test-position sets using KFold indexing (illustrated here with one fold for clarity; five folds are used in Table 1, Supplementary Table 6, and Supplementary Figure 10). Variants are then assigned based on the positions of all mutations they carry: variants mutated exclusively at train positions are assigned to train, variants mutated exclusively at test positions are assigned to test, and variants carrying mutations from both position sets are excluded to prevent positional overlap. This design ensures that the model is evaluated only on mutation sites never seen during training, providing a more realistic estimate of generalization to novel mutational positions

A primary, yet often overlooked, obstacle in applying PLMs to viral data is the phenomenon of “homology leakage”. Due to the extreme sequence similarity within viral families, standard random training-test splits often allow models to achieve deceptively high accuracy by memorizing site-specific fitness effects rather than learning generalizable biophysical rules. This results in performance metrics that fail to translate into predictive utility for novel, emerging variants. These concerns are corroborated by recent evidence demonstrating that pretrained PLMs can inflate performance scores in paired biological tasks through the memorization of training sequences [46]. Critically, such models have been shown to fail in generalizing to the effect of point mutations on binding affinities, highlighting a fundamental gap in current evaluation protocols. To address this directly, we introduce a position-stratified splitting strategy that partitions mutation sites — rather than sequences — into disjoint train and test sets, ensuring the model is never evaluated on positions encountered during training (Fig. 1). Unlike random splits, where the same mutated positions routinely appear in both partitions and artificially inflate performance estimates, position-stratified splits provide a more stringent test of generalization to unseen mutational sites.

Here, we present **PRIME** (Protein Representation Inference for Mutation Evaluation), a framework that attempts to address these limitations by combining domain-specific fine-tuning of state-of-the-art PLMs with a rigorous position-stratified validation protocol. Our approach demonstrates three key advances: (1) we show that fine-tuning is essential for models to predict phenotypes at previously “unseen” mutational sites; (2) we demonstrate that neural networks built upon these specialized embeddings achieve meaningful predictive power on stratified benchmarks where base foundation models fail; and (3) we show that embedding-based functional clustering can identify host tropism patterns and provide a complementary perspective on functional similarity among bat coronavirus sequences relative to traditional phylogenetic approaches. PRIME complements emerging proactive surveillance methods by establishing a rigorous evaluation standard — position-stratified validation — that can serve as a benchmark for any PLM-based framework operating in high-homology viral sequence space.

## Results

### Benchmark data collection for viral phenotype prediction

**Fig. 2:**
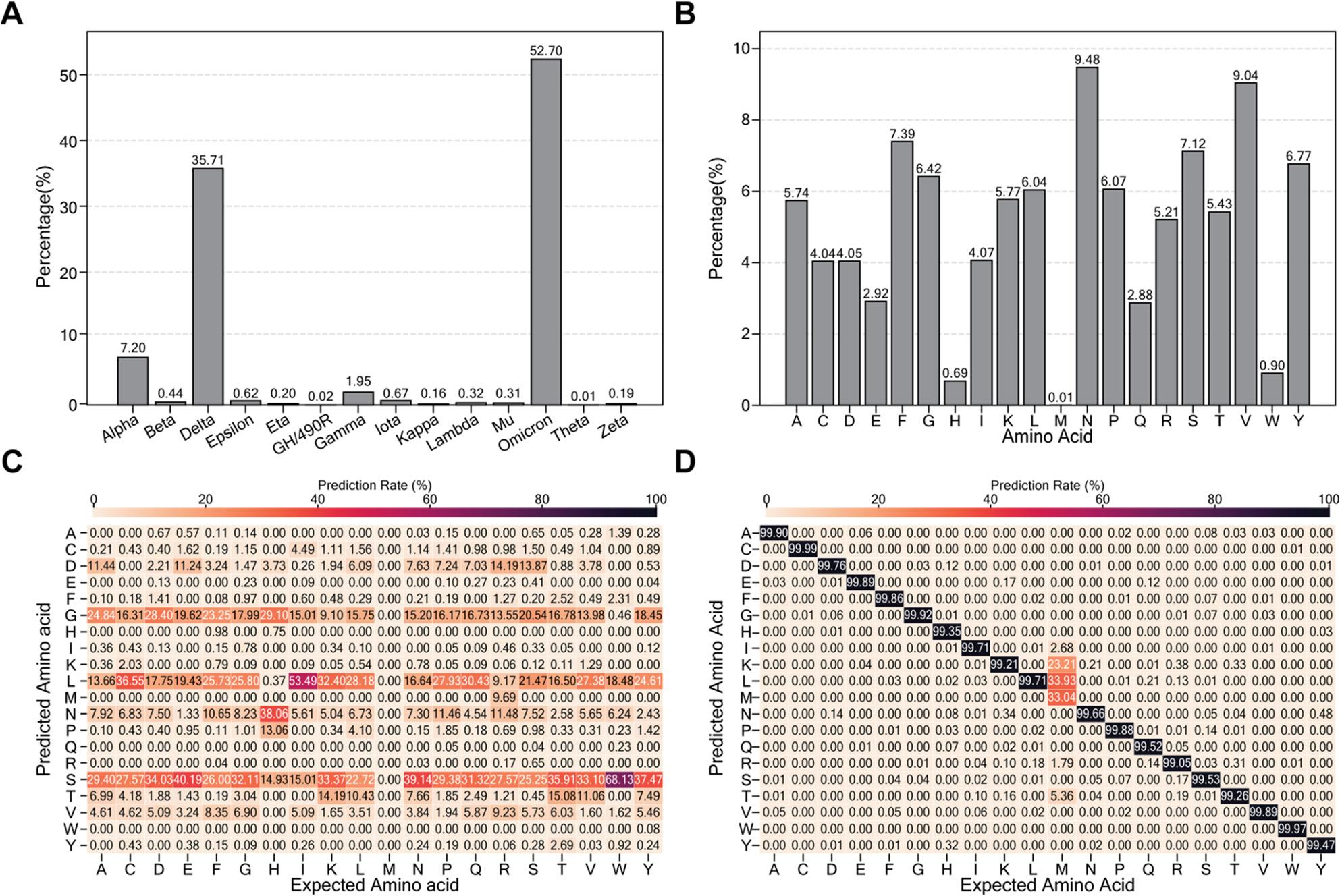
SARS-CoV-2 RBD sequence analysis and language model performance comparison. **A** Distribution of viral lineages in the outbreak dataset (n = 347,432 sequences), showing predominance of Omicron (52.70%) and Delta (35.71%) variants. **B** Amino acid frequency distribution within RBD sequences, highlighting variability in representation from most common (Asparagine, 9.48%) to rare occurrences (Methionine, 0.01%). **C** Confusion matrix of the non-fine-tuned ESM-2 8M model amino acid prediction accuracy. Diagonal values represent correct prediction rates for each amino acid, with overall accuracy of 5.06%. **D** Confusion matrix for the PRIME fine-tuned ESM-RBD (ESM-2 8M) model after 100 epochs, demonstrating a 19-fold improvement in sequence understanding with an overall masked language modeling accuracy of 96.33%

We collected and curated three complementary sequencing datasets, “outbreak”, “DMS” and “betaCov”, to comprehensively analyze RBD sequences. The **outbreak dataset** comprises 347,432 unique RBD sequences curated from GISAID [47] and the Sequence Read Archive (SRA) [48] as of February 3rd, 2023, with all duplicates and sequences containing ambiguous amino acids removed. This dataset represents viral sequences under real-time selection pressure during the SARS-CoV-2 pandemic, spanning all major SARS-CoV-2 lineages, with Omicron (52.70%), Delta (35.71%), and Alpha (7.20%) variants accounting for 95.61% of the sequences (Fig. 2A). Amino acid distribution analysis revealed representation of all 20 canonical amino acids, though with notable variability—Leucine (9.48%), Valine (9.04%), and Serine (7.12%) were most abundant, while Methionine appeared in only 0.01% of positions and fewer than 1% of all sequences (Fig. 2B). Sequence length distribution was highly uniform, with 99.75% of the sequences consisting of 223 amino acids.

For evaluating model performance in predicting functional properties, we utilized the **DMS dataset** containing 116,257 unique RBD sequences with measured ACE2 binding affinities and 105,525 unique RBD sequences with quantified expression levels from yeast in vitro experiments [36].

To assess cross-species coronavirus predictions, we assembled the **betaCov dataset** containing 75 RBD sequences from diverse Sarbecovirus members, including SARS-CoV-2 (Wuhan-HU-1 strain [49], RaTG13v (a bat coronavirus reportedly closely related to SARS-CoV-2 [50]), SARS-CoV-1 [51], MERS-CoV [52], Pangolin-CoV [53], and various bat coronaviruses used in a recent survey of sarbecovirus ACE2 binding [36]. This dataset represents RBD sequences targeting different mammalian hosts, including bat, pangolin, civet, camel, and human (**Supplementary Table 1**), with some SARS-CoV-1 and MERS-CoV sequences demonstrating cross-species receptor binding.

### Scaling and fine-tuning overcome the generalization gap

To evaluate the predictive limits of Protein Language Models (PLMs) in the context of high sequence homology, we benchmarked five PLM architectures spanning two architectures: ESM-2 [40] (8 M, 150 M, 650 M) and ESM-C [52] (300 M, 600 M), under both random and position-stratified regimes. To address “homology leakage”, we implemented **position-stratified cross-validation**, where all mutations at specific residue positions are held out from the training set to measure genuine biological generalization.

We first assessed whether domain-specific fine-tuning could improve the model’s representation of the RBD sequence space. The original ESM-2 8 M model, without fine-tuning, achieved only 5.06% accuracy in masked language modeling (MLM) tasks. After 100 epochs of fine-tuning, the resulting **ESM-RBD** model achieved 96.33% MLM accuracy—a 19-fold improvement (Fig. 2C and D).

We compared this to an in-house BERT [54]-based model (**BERT-RBD**) trained from scratch on only RBD sequences. While BERT-RBD reached 94.09% MLM accuracy (**Supplementary Fig. 1A**,** 1B**), it remained inferior to the fine-tuned ESM models in capturing rare biochemical signatures. Most notably, ESM-RBD predicted Methionine with 33.04% accuracy, whereas BERT-RBD failed to predict it entirely. This highlights a key advantage of the PRIME framework: fine-tuning a pre-trained foundation model leverages broad, evolutionarily informed representations of protein sequence patterns that are difficult to recover when training from scratch on narrow, pathogen-specific datasets.

The necessity of both scale and fine-tuning becomes most apparent when confronting the generalization gap in phenotype prediction (Table 1). Our results demonstrate that performance metrics from random splits are highly inflated due to data leakage. For the **ESM-C 600M** model, using mean embedding across all amino acid residues in the RBD, a random split yielded a deceptively high R^2^ = 0.7711 for binding affinity. However, under the rigorous position-stratified split, the base foundation model (without domain-specific fine-tuning) failed to provide any predictive signal, resulting in a negative correlation coefficient (R^2^=-0.1050) (Table 1).

**Table 1:** Benchmarking PRIME across different model scales and validation regimes for mutated RBD binding and expression

Model	EmbedMethod	Fine-tuned	Random Split				Position-Stratified Split			
			Binding		Expression		Binding		Expression	
			R²	RMSE	R²	RMSE	R²	RMSE	R²	RMSE
ESM-2 8M	CLS	×	0.5876 ± 0.07	1.2191 ± 0.10	0.5984 ± 0.07	0.6272 ± 0.05	-0.2018 ± 0.04	1.9452 ± 0.04	-0.0324 ± 0.05	1.0916 ± 0.02
		✓	0.5897 ± 0.03	1.2194 ± 0.04	0.5302 ± 0.03	0.6808 ± 0.02	-0.2294 ± 0.03	1.9653 ± 0.03	-0.1928 ± 0.02	1.1756 ± 0.01
	Mean	×	0.6794 ± 0.02	1.0777 ± 0.04	0.6576 ± 0.04	0.5807 ± 0.03	0.0248 ± 0.01	1.7519 ± 0.01	0.0967 ± 0.02	1.0221 ± 0.01
		✓	0.6225 ± 0.07	1.1659 ± 0.10	0.5545 ± 0.07	0.6613 ± 0.05	0.0071 ± 0.05	1.7688 ± 0.06	0.1013 ± 0.06	1.0213 ± 0.03
ESM-2 150M	CLS	×	0.5788 ± 0.14	1.2167 ± 0.22	0.5409 ± 0.08	0.6709 ± 0.06	-0.2449 ± 0.06	1.9777 ± 0.03	-0.0754 ± 0.04	1.1169 ± 0.02
		✓	0.5836 ± 0.28	1.1639 ± 0.40	0.3354 ± 0.45	0.7664 ± 0.26	-0.2675 ± 0.07	2.0022 ± 0.04	-0.3805 ± 0.04	1.2480 ± 0.02
	Mean	×	0.6713 ± 0.13	1.0693 ± 0.22	0.6131 ± 0.07	0.6150 ± 0.06	-0.0695 ± 0.03	1.8359 ± 0.02	0.0195 ± 0.03	1.0649 ± 0.02
		✓	0.7461 ± 0.08	0.9480 ± 0.15	0.6693 ± 0.04	0.5704 ± 0.03	-0.0080 ± 0.00	1.7863 ± 0.01	0.1039 ± 0.04	1.0165 ± 0.02
ESM-2 650M	CLS	×	0.7694 ± 0.13	0.8757 ± 0.27	0.7139 ± 0.13	0.5171 ± 0.12	-0.1591 ± 0.04	1.9063 ± 0.02	-0.0004 ± 0.03	1.0781 ± 0.01
		✓	0.7584 ± 0.11	0.9104 ± 0.22	0.6758 ± 0.10	0.5582 ± 0.09	-0.0399 ± 0.06	1.8084 ± 0.06	-0.0175 ± 0.06	1.0869 ± 0.03
	Mean	×	0.7981 ± 0.13	0.8091 ± 0.28	0.7337 ± 0.15	0.4942 ± 0.14	-0.0865 ± 0.04	1.8488 ± 0.03	-0.0103 ± 0.03	1.0807 ± 0.01
		✓	0.8189 ± 0.10	0.7798 ± 0.22	0.7354 ± 0.11	0.4999 ± 0.10	0.0588 ± 0.04	1.7200 ± 0.03	0.0779 ± 0.04	1.0336 ± 0.02
ESM-C 300M	CLS	×	0.5869 ± 0.15	1.2032 ± 0.23	0.6026 ± 0.08	0.6230 ± 0.06	-0.1954 ± 0.06	1.9366 ± 0.04	-0.0313 ± 0.04	1.0924 ± 0.02
		✓	0.7647 ± 0.08	0.9104 ± 0.16	0.6678 ± 0.07	0.5691 ± 0.06	0.0019 ± 0.05	1.7766 ± 0.04	0.0916 ± 0.02	1.0265 ± 0.02
	Mean	×	0.7155 ± 0.07	1.0075 ± 0.13	0.6546 ± 0.04	0.5830 ± 0.03	-0.0162 ± 0.01	1.7874 ± 0.02	0.0647 ± 0.02	1.0410 ± 0.01
		✓	0.8057 ± 0.06	0.8278 ± 0.14	0.7165 ± 0.06	0.5261 ± 0.05	0.1949 ± 0.03	1.5918 ± 0.03	0.2313 ± 0.01	0.9449 ± 0.01
ESM-C 600M	CLS	×	0.6616 ± 0.12	1.0863 ± 0.22	0.6030 ± 0.09	0.6219 ± 0.07	-0.1106 ± 0.02	1.8726 ± 0.00	-0.0167 ± 0.03	1.0823 ± 0.01
		✓	0.7552 ± 0.07	0.9308 ± 0.15	0.5388 ± 0.21	0.6577 ± 0.15	0.1165 ± 0.06	1.6616 ± 0.05	0.1478 ± 0.01	0.9922 ± 0.01
	Mean	×	0.7355 ± 0.12	0.9525 ± 0.23	0.6610 ± 0.08	0.5738 ± 0.07	-0.0973 ± 0.03	1.8558 ± 0.02	0.0666 ± 0.02	1.0398 ± 0.02
		✓	0.8346 ± 0.07	0.7560 ± 0.17	0.7342 ± 0.06	0.5087 ± 0.06	0.2120 ± 0.03	1.5745 ± 0.02	0.2479 ± 0.02	0.9334 ± 0.02
One-Hot Mean			0.1136 ± 0.03	1.7930 ± 0.03	0.0617 ± 0.02	0.9625 ± 0.01	-0.2397 ± 0.01	1.9754 ± 0.00	-0.1774 ± 0.01	1.1675 ± 0.00

Performance is reported as the coefficient of determination (R2) and Root Mean Square Error (RMSE), with ± values representing the standard deviation across random seeds seen in Supplementary Table 6. Models used either mean embeddings, where sequence-level feature was derived by calculating the mean amino acid representation (average of all site-specific embeddings) across the entire sequence domain, or the standard CLS token embedding as the sequence-level representation. If the model is not designated as fine-tuned, the base ESM model is used as the embedder

Domain-specific fine-tuning on the outbreak dataset significantly restored predictive power in the stratified context. For the ESM-C 600 M model, fine-tuning improved stratified binding affinity R^2^ from no measurable signal (-0.1050) to a modest predictive value of 0.1823 ± 0.2149 using mean amino acid representation and 0.2087 ± 0.1451 using sequence level CLS embeddings. Similar improvement was found in fine-tuned ESM-C models under a stratified expression split (Table 1). These findings demonstrate that while foundation models are essential starting points, pathogen-specific adaptation is needed to capture the nuanced biophysical constraints necessary for prospective biosurveillance.

### PLM scale and fine-tuning resolve viral lineages and diversity

**Fig. 3:**
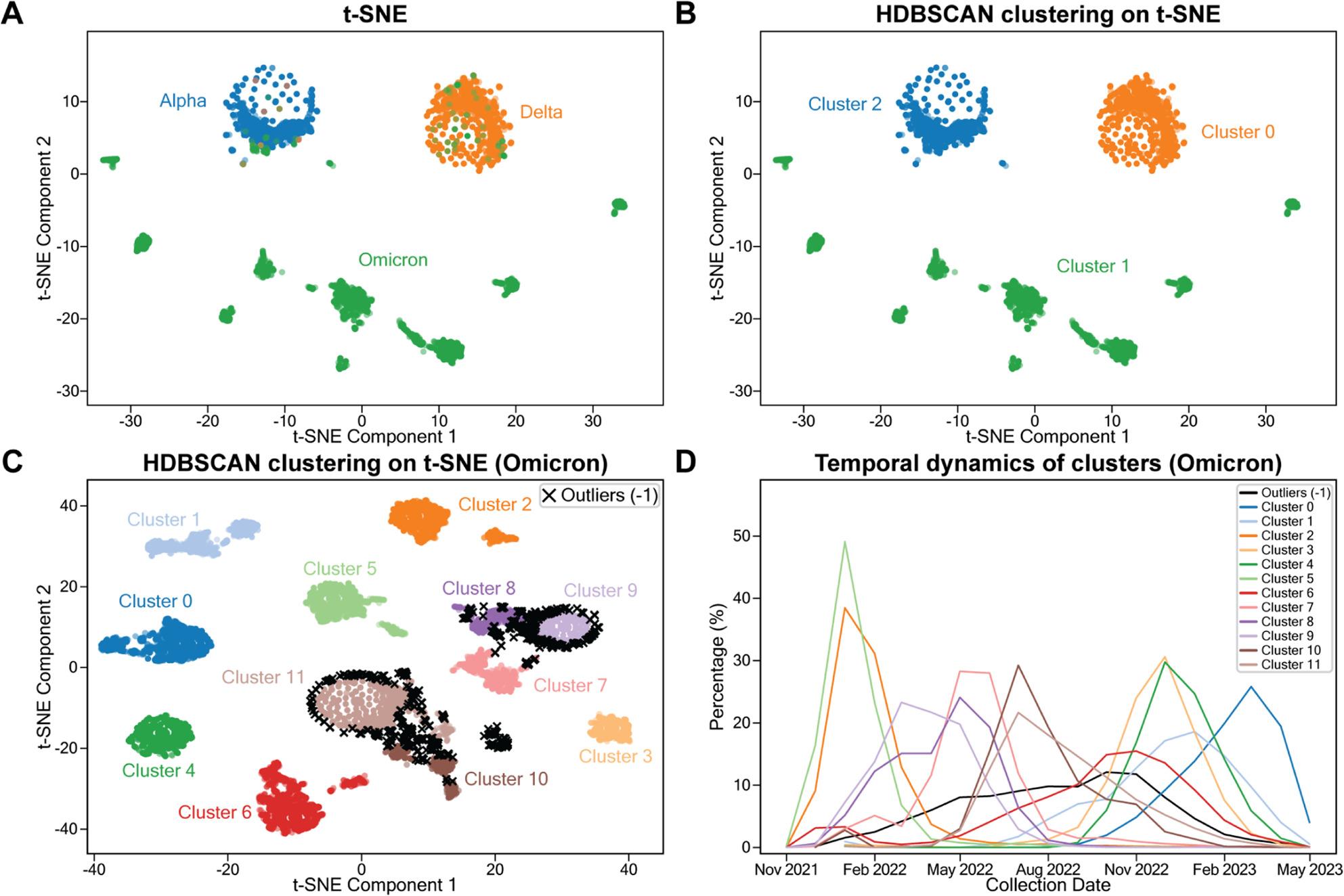
Fine-tuned ESM-RBD (ESM-2 8M) embeddings resolve viral lineages and capture temporal sub-population dynamics. **A**, **B**: t-SNE visualization and HDBSCAN clustering of sequence-level latent representations for Alpha (blue), Delta (orange), and Omicron (green) lineages. Fine-tuning enables the model to effectively partition major lineages with an Adjusted Rand Index of 0.98. **C** HDBSCAN clustering applied to t-SNE representations of all available Omicron sequences (n = 160,016) reveals 12 clusters (0-11) with some outliers (-1), demonstrating significant diversity within the Omicron lineage. **D** Temporal dynamics of Omicron clusters from November 2021 to May 2023, showing the emergence and succession of specific sub-lineages (e.g., Cluster 7) during the pandemic

We evaluated whether the learned representations of the fine-tuned ESM-RBD model could enable rapid identification of evolutionary relationships and distinguish major SARS-CoV-2 lineages based solely on RBD sequence embeddings. To address the imbalance in lineage abundance, we randomly downsampled the Omicron (*n* = 160,016) and Delta (*n* = 109,448) sequences to match the number of Alpha lineage sequences (*n* = 22,075 per lineage). Visualization of these sequence-level latent representations using t-SNE produced three distinct clusters corresponding to the Alpha, Delta, and Omicron lineages (Fig. 3A). HDBSCAN clustering of these representations showed high agreement with established lineage assignments, with Cluster 0 corresponding predominantly to Delta, Cluster 1 to Omicron, and Cluster 2 to Alpha (Fig. 3B). We validated the robustness of this organization through nine iterations of random downsampling (**Supplementary Fig. 2**) and across a range of t-SNE perplexities, where optimal separation was achieved at a perplexity of 750 (Adjusted Rand Index [ARI] = 0.98) (**Supplementary Fig. 3–5**).

To further explore diversity within the rapidly evolving Omicron lineage, we applied our clustering approach to the complete set of Omicron sequences (*n* = 160,016), revealing 12 distinct sub-clusters (Fig. 3C). Tracking the temporal distribution of these clusters from November 2021 to May 2023 demonstrated clear patterns of variant succession, such as the early dominance of Clusters 2 and 5 followed by the rise of Cluster 7 in May 2022 (Fig. 3D). These sub-clusters showed significantly higher correspondence with established Pango lineage classifications—achieving > 95% purity for lineages such as BA.1.1* and XBB.1.5—compared to MSA-based approaches (**Supplementary Fig. 6**,** Supplementary Table 2**).

Systematic comparison demonstrated that domain-specific fine-tuning is valuable for high-resolution lineage discrimination. While the non-fine-tuned ESM baseline could separate major lineages, it exhibited lower resolution within the Omicron lineage, identifying only 10 clusters compared to the 12 identified by ESM-RBD (**Supplementary Fig. 7**). Furthermore, ESM-RBD outperformed MSA-based clustering (**Supplementary Fig. 8**), which failed to capture fine-grained evolutionary relationships and yielded lower overall clustering metrics (ARI = 0.90 for MSA vs. ARI = 0.98 for ESM-RBD) (**Supplementary Fig. 9**). These results indicate that fine-tuning enables the model to identify functionally and evolutionarily relevant sequence patterns that are obscured by general-purpose models without fine-tuning and traditional MSA-based methods do not provide this type of resolution.

### Optimization of neural architectures for real-time deployment

To identify which components drive predictive performance, we conducted a systematic ablation across four neural network architectures: fully connected networks (FCN), graph convolutional networks (GCN) [55], bidirectional long short-term memory networks (BLSTM) [56], BERT-RBD), two learning rates, and both frozen and fine-tuned encoder conditions, using base ESM and ESM-RBD initializations (**Supplementary Tables 3 and 4**). This design allows us to isolate the contribution of embedding quality from that of the downstream regressor architecture. The FCN architecture with sequence-level (CLS) representations achieved superior predictive performance across both functional properties while maintaining a significantly smaller parameter footprint (**Supplementary Tables 3 and 4**).

**Fig. 4:**
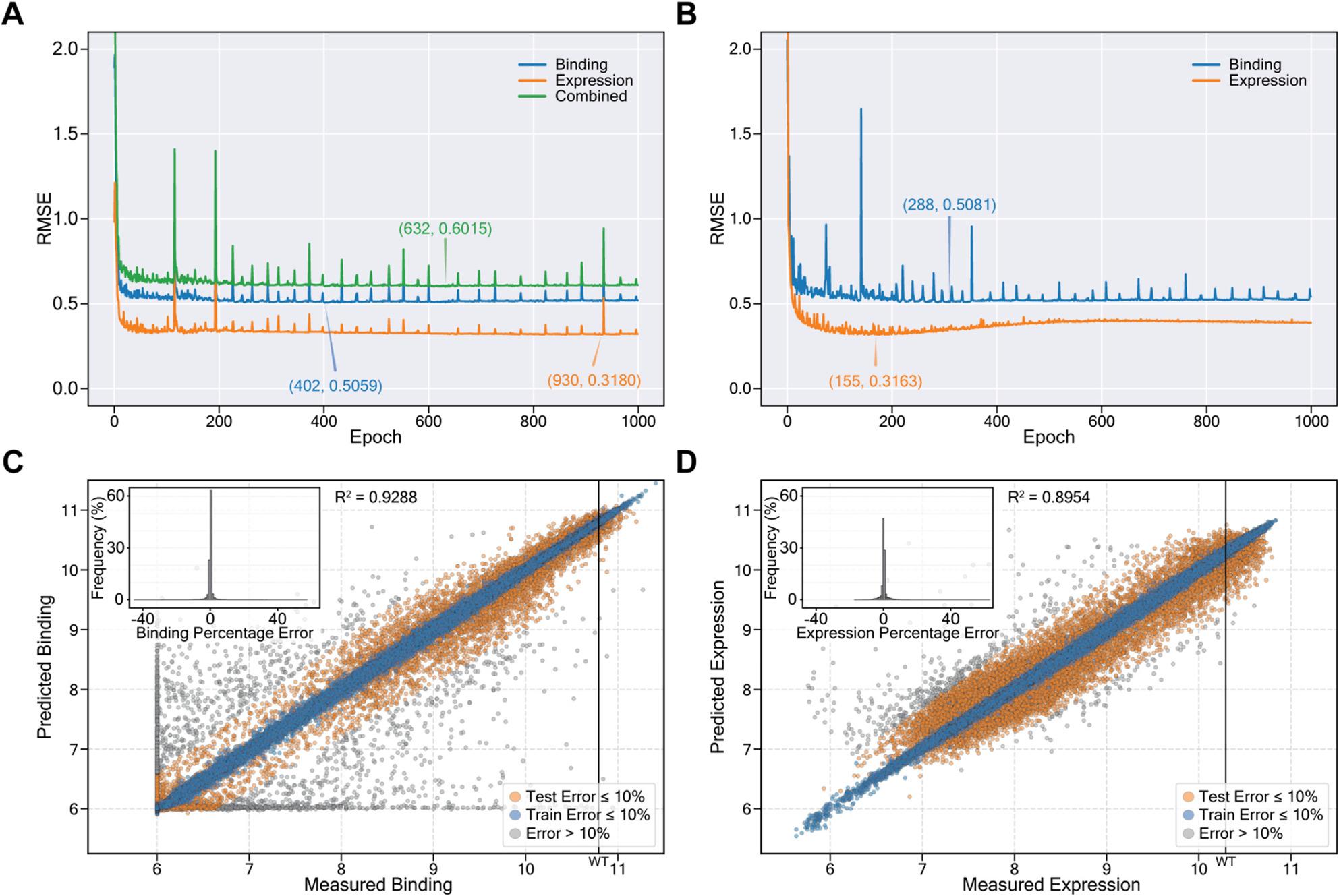
Multi-task architecture optimization and fitting capacity for RBD phenotypes. **A** Training progression for the multi-task ESM-RBD-FCN (ESM-2 8M) model showing RMSE values for binding (blue), expression (orange), and combined loss (green). The model achieved lowest validation RMSE at epoch 402 for binding (RMSE = 0.5059) and epoch 930 for expression (RMSE = 0.3180), with optimal combined performance at epoch 632 (RMSE = 0.6015). **B** Performance comparison of single-task models optimized for binding (blue, optimal at epoch 288, RMSE = 0.5081) and expression (orange, optimal at epoch 155, RMSE = 0.3163), demonstrating that the multi-task approach maintains performance while halving computational overhead. **C**, **D** Correlation between experimentally measured and predicted scores for binding affinity (R2=0.9288) and protein expression (R2=0.8954) under a random-split regime. Insets: Error distributions show that over 95% of predictions fall within 5% of experimental values. Outliers (>10% error, gray) predominantly correspond to residues at functionally critical sites such as N501

We further evaluated the impact of multi-task learning by training a single FCN to simultaneously predict ACE2 binding affinity and protein expression levels using the DMS dataset [36]. This multi-task approach reduced total computational requirements by approximately 50% without a significant loss in accuracy compared to single-task models (Fig. 4A and B). Training dynamics showed stable convergence, with optimal performance for binding and expression prediction achieved at epoch 402 and epoch 155, respectively (Fig. 4A and B, **Supplementary Tables 3**, and **Supplementary Table 4**).

While the correlation coefficients observed in random-split evaluations (R^2^ = 0.9288 for binding; R^2^ = 0.8954 for expression) are inflated by sequence homology, the error analysis provides critical insight into the model’s high-fidelity representation of known mutational space (Fig. 4C and D). Over 95% of predictions fell within a 5% error threshold of experimental measurements, with error distributions tightly centered around zero. Systematic analysis of outliers revealed that residues with errors exceeding 10% were predominantly located at functionally critical sites, such as N501, which are known to exhibit complex, non-linear effects on ACE2 binding (**Supplementary Table 5**) [57]. This indicates that the FCN architecture effectively captures the majority of the biochemical landscape, but systematic failures pinpoint specific residues where more advanced biophysical modeling is required.

### ESM-RBD embeddings identify candidate bat coronavirus sequences for experimental follow-up

**Fig. 5:**
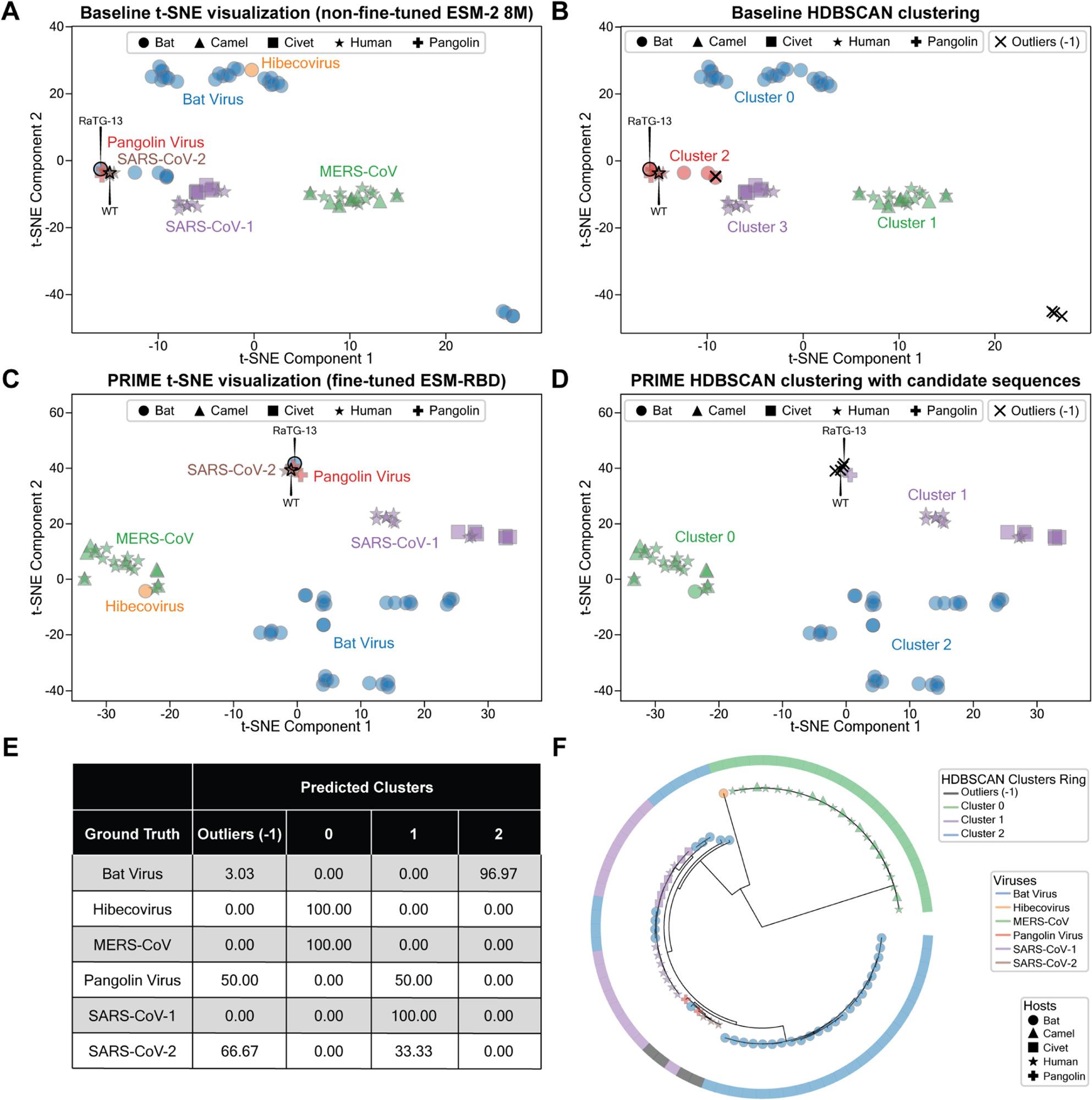
Domain-specific fine-tuning resolves host tropism patterns and zoonotic signatures among betacoronaviruses. **A**, **B** Baseline representation: t-SNE visualization and HDBSCAN clustering of 75 betacoronavirus RBD sequences using the non-fine-tuned ESM-2 8M model. Baseline embeddings exhibit limited resolution, failing to clearly delineate functional groups or identify outliers with zoonotic potential. **C**, **D** PRIME representation: t-SNE visualization and HDBSCAN clustering using the fine-tuned ESM-RBD (ESM-2 8M) model. **E** Cross-tabulation of host annotations versus predicted clusters as seen in 5D. PRIME identifies 3.03% of bat coronaviruses that cluster with known human-infective lineages (Cluster 1), suggesting functional similarity to human-infective strains in embedding space; external experimental validation would be required to assess zoonotic risk. **F** Maximum-likelihood phylogeny of the betacoronavirus dataset

To evaluate whether fine-tuned PLMs can detect functional signatures associated with diverse host spillover risk, we analyzed the latent representations of 75 betacoronavirus RBD sequences spanning diverse host species (**Supplementary Table 1**). We first established a baseline using the non-fine-tuned ESM model, where dimensionality reduction and HDBSCAN [58] clustering of embeddings exhibited limited resolution and failed to clearly separate groups (Fig. 5A and B). In contrast, clustering of the fine-tuned ESM-RBD embeddings identified three distinct clusters that more effectively recapitulated established taxonomic and functional relationships (Fig. 5C and D). Cluster 0 comprised MERS-CoV and hibecovirus sequences, while Cluster 1 contained SARS-CoV-1, SARS-CoV-2, and pangolin coronaviruses. Cluster 2 consisted primarily of bat coronaviruses.

Qualitative comparison of the t-SNE projections between the baseline and fine-tuned models suggests improved cluster separation following fine-tuning; we note that this comparison is visualization-supported and exploratory rather than a formal quantitative metric, as t-SNE distances are not directly interpretable (Fig. 5A and C). This value was calculated by comparing the maximum range of the t-SNE axis components, where the fine-tuned model projected sequence into a broader, more discriminative embedding space. Quantitative analysis of this refined space revealed high clustering consistency with host annotations, achieving 100% accuracy for MERS-CoV, hibecovirus, and pangolin coronavirus sequences (Fig. 5E). Significantly, 3.03% of sequences annotated as bat coronaviruses clustered with the zoonotic SARS-CoV-1/SARS-CoV-2 group (Cluster 1) rather than the primary bat virus cluster (Fig. 5E). These sequences cluster in closer proximity to known human-infective strains in embedding space (Fig. 5D), suggesting functional similarity that warrants further experimental investigation, e.g. ACE2 binding compatibility assays. We note that clustering proximity alone does not establish spillover potential, and external validation would be required to assess true zoonotic risk.

We compared this approach to a traditional maximum likelihood phylogenetic tree (Fig. 5F). The phylogeny and clustering method corroborated the broad taxonomic groupings— notably placing MERS-CoV in a distinct, distantly related clade. However, PRIME’s embedding space uniquely focuses on the biophysical similarity between human pathogens and select bat coronaviruses. The two methods offer complementary perspectives: PRIME’s embedding space uniquely emphasizes the functional similarity or representation geometry whereas the phylogeny emphasizes evolutionary relationships. These methods can be used in combination for improved biosurveillance.

PRIME may offer a complementary perspective for evaluating functional similarity to human-infective strains: the embedding-based clustering places these specific bat viruses closer to the SARS-CoV-1/SARS-CoV-2 group (Fig. 5D) than to the primary bat-associated cluster, whereas phylogeny reflects evolutionary proximity without directly capturing this functional distinction. Furthermore, the embedding analysis showed remarkable robustness to sequence divergence. The hibecovirus sequence, which failed standard composition tests during phylogenetic reconstruction, was seamlessly incorporated and accurately clustered by our model (Fig. 5C). Although these observations are based on a limited dataset (*n* = 75), they suggest that fine-tuned PLM embeddings may capture biologically relevant structural and functional constraints and could provide a complementary perspective for assessing spillover potential. Further validation on larger datasets will be necessary to confirm these trends.

## Discussion

The central challenge of applying protein language models to viral pathogens like SARS-CoV-2 is the deceptive nature of sequence homology. As highlighted by our benchmarking of random vs. position-stratified splits, standard validation protocols often lead to “homology leakage,” where models achieve high accuracy (R2 > 0.90) by memorizing site-specific fitness rather than learning the underlying biophysical rules. PRIME addresses this by establishing the importance of **position-stratified validation**.

While previous studies have noted that PLM-based models struggle to generalize to point mutations in protein-protein interactions [46], our results suggest that this is not an inherent limitation of the architecture, but rather a consequence of insufficient domain adaptation and improper validation splitting. By implementing position-stratified splits, we demonstrate that domain-specific fine-tuning can indeed recover a meaningful predictive signal (R^2^ ≈ 0.23 for **ESM-C 600M**) for binding affinity on entirely unseen sites. Our findings suggest that base models fail to generalize to novel mutational sites (R^2^ < 0), whereas domain-specific fine-tuning on large-scale “outbreak” datasets lead to improved predictive performance.

While domain-specific fine-tuning consistently improves stratified performance relative to base models — which show no predictive signal (R² < 0) — the resulting R² values of approximately 0.18–0.23 for binding affinity on unseen mutational sites reflect a meaningful but modest level of position-stratified generalization — which, while not strictly zero-shot given the domain-specific fine-tuning applied, represents a meaningful test of the model’s ability to extrapolate to entirely unseen mutational positions. These results should not be interpreted as a resolution of the generalization challenge, but rather as evidence that fine-tuning on large-scale outbreak data provides a foundation for genuine prospective prediction. True generalization to entirely novel mutational contexts remains an open problem, likely requiring richer structural priors, larger domain-specific corpora, or multi-modal training signals beyond sequence alone. We therefore frame PRIME’s stratified benchmark as a more honest baseline from which future work can measure real progress.

Our systematic evaluation reveals that neither model scale nor pre-training alone is sufficient for pathogen-specific accuracy. General-purpose PLMs, trained on “tree of life” scale data with limited coverage of viral sequence variations, often lack the resolution to distinguish the subtle variations that determine viral host specificity or immune escape. We found that even state-of-the-art architectures like ESM-C 600 M require fine-tuning to move from negative to positive correlation on stratified tasks. This suggests that fine-tuning allows the model to capture nuanced biophysical constraints—such as those at functionally critical sites like N501—that are otherwise invisible to base foundation models. Furthermore, our analysis confirms that mean pooling consistently outperforms CLS-token representations across all five ESM model scales under five-fold cross-validation. This advantage likely reflects the nature of the RBD itself: phenotypic properties such as ACE2 binding affinity are determined by the collective contribution of residues distributed across the domain rather than any single site. Mean pooling aggregates these distributed site-specific signals into a single representation, whereas the CLS token — originally designed to capture global sequence context during pre-training — may not optimally encode this kind of position-averaged biochemical information when repurposed as a downstream regression feature. The robustness of these findings across three random seeds and multiple fold assignments (Supplementary Table 6, Supplementary Fig. 10) further supports the stability of this conclusion.

PRIME’s embedding-based clustering and traditional phylogenetic methods address related but distinct questions: phylogeny aims to reconstruct evolutionary history, whereas PRIME’s embedding space captures functional and biochemical similarity that may not strictly follow neutral genetic drift. These approaches are therefore best viewed as complementary tools for biosurveillance. Both methods successfully separated MERS-CoV and hibecoviruses from SARS-associated viruses, consistent with their divergent receptor usage (DPP4 vs. ACE2). Where phylogeny provides evolutionary context and ancestral relationships, PRIME may offer additional resolution for identifying sequences with convergent functional properties — such as shared receptor binding profiles — regardless of phylogenetic distance. By focusing on features related to host receptor interactions rather than neutral sequence similarity, PRIME can help highlight mutations that may be functionally relevant even when phylogenetic signal is limited or misleading.

The betacoronavirus analysis should be interpreted with appropriate caution. The dataset is small (*n* = 75 RBD sequences) and host labels are coarse, and the boundary separating bat-associated sequences from human-infective clusters in the embedding space is conceptual rather than quantitatively derived. While PRIME identified 3.03% of bat coronavirus sequences clustering near known human-infective strains, this observation reflects functional similarity in embedding space and does not constitute evidence of elevated zoonotic potential. These sequences are best understood as candidates for experimental prioritization — for example, for ACE2 binding assays — rather than confirmed high-risk variants. Future work with larger, more diverse datasets and paired experimental validation would be needed to determine whether embedding-space proximity is genuinely predictive of host-jump capacity.

Notably, PRIME may provide a more informative and functional assessment of zoonotic risk than baseline foundation models. In the non-fine-tuned baseline (Fig. 4B), several bat coronavirus sequences appeared proximal to the SARS-CoV-1 group. However, following domain-specific fine-tuning, PRIME re-assigned these sequences to the primary bat-associated Cluster 2 (Fig. 4D). This suggests that PRIME may better differentiate between neutral genetic homology and the specific functional requirements needed for efficient human ACE2 binding.

While no bat viruses were directly assigned to the human-pathogen Cluster 1, PRIME identified 3.03% of bat coronavirus sequences that exhibited high functional similarity to human pathogens as outliers (Cluster − 1), pinpointing specific candidates within the “human-infective” embedding space (Fig. 5D and E). This group includes RaTG-13, which occupies a unique position in the embedding space near the hypothetical zoonotic boundary, reflecting its high similarity to human pathogens while accurately signaling its functional distinctness. Similarly, PRIME classified 50% of pangolin-derived sequences within Cluster 1 (Fig. 5D and E), aligning with experimental reports that specific pangolin-CoV strains exhibit high affinity for human ACE2 [59]. By focusing on “human vs. non-human” functional profiles rather than over-interpreting evolutionary distance, PRIME identifies candidates for experimental prioritization based on their embedding-space proximity to human-infective strains, offering a potentially complementary signal for early-stage surveillance.

The computational efficiency of PRIME suggests potential for near real-time deployment. Embedding-based clustering scales significantly better than maximum likelihood phylogenetics, allowing for the analysis of thousands of sequences in minutes. Additionally, the framework’s robustness to sequence divergence—demonstrated by the seamless integration of highly divergent hibecoviruses—makes it ideal for monitoring rapidly evolving pathogens that challenge traditional alignment tools.

PRIME is designed as an analytical tool to assess phenotypic risk from sequence data rather than a generative model for sequence design. All sequence data used in this study are drawn from publicly accessible databases (GISAID, GenBank) under their respective data access agreements. Model weights for the ESM-RBD model (8 M only) and code are released under the MIT license with the intent of supporting legitimate biosurveillance research. Limitations on GitHub repo size and file size prevent the addition of other weights. As PLMs become more capable, integrating built-in guardrail, such as restricting applications to surveillance rather than design contexts, and ensuring compliance with institutional biosafety review, will be essential for their responsible use and deployment.

## Conclusions

PRIME establishes a scalable framework for pathogen biosurveillance that prioritizes true biological generalization over deceptive pattern memorization. By combining state-of-the-art model scale with domain-specific fine-tuning and stratified validation, it provides a framework that may assist in prioritizing sequences for experimental follow-up in the context of viral surveillance.

## Methods

### Data collection and curation

Three distinct datasets were utilized to develop and validate the PRIME framework:

#### Outbreak dataset

This dataset comprises 347,432 unique SARS-CoV-2 RBD sequences (amino acids 319–541of the spike protein) curated from GISAID and SRA as of February 3rd, 2023. All sequences containing ambiguous amino acids or duplicates were removed to ensure data integrity.

#### DMS dataset

For phenotype prediction, we utilized experimental Deep Mutational Scanning (DMS) data containing 116,257 unique RBD sequences with measured ACE2 binding affinities and **105**,**525 sequences** with quantified protein expression levels from in vitro yeast display experiments.

#### BetaCov dataset

To assess cross-species generalization, we assembled **75 RBD sequences** from diverse Sarbecovirus members, including SARS-CoV-1, SARS-CoV-2, Pangolin virus, Hibecovirus, and various bat viruses were sourced from a recent survey of sarbecovirus ACE2 binding [36]. In total, there are 63 GenBank/NCBI sequences and 3 GISAID sequences, accessible through *doi*: 10.55876/gis8.250709on. MERS sequences were manually retrieved by querying the accession number ALA49374.1 using NIH’s BLASTp. From the top few hundred results, we carefully filtered out duplicates and identified relevant host species. The complete list of this dataset is presented in **Supplementary Table 1**.

### Model architecture and scale

We systematically benchmarked five protein language model (PLM) architectures to evaluate the impact of parameter scale:

#### ESM-2 family

We utilized the 8 M (6 layers), 150 M (30 layers), and 650 M (33 layers) parameter models as baseline foundations.

#### ESM-C family

We incorporated the state-of-the-art ESM-C 300 M and ESM-C 600 M models to assess the benefits of modern architecture design on viral representation learning.

#### BERT-RBD

A BERT-based model was trained from scratch with a 320-dimensional embedding space as a domain-specific control.

### Domain-specific fine-tuning

Fine-tuning was performed using a Masked Language Modeling (MLM) objective. For the ESM-RBD 8 M model, 15% of amino acids were randomly masked, and the model was tasked with predicting these residues based on contextual information. Fine-tuning was conducted over 100 epochs at a learning rate of 1 × 10^− 5^. For scaling tests, fine-tuning was performed over 15 epochs at the same learning rate. Fine-tuning used the AdamW optimizer with β1 = 0.9, β2 = 0.999 and no warm-up schedule. The effective batch size was 512, or 64 sequences per GPU across eight A100 GPUs using Distributed Data Parallel. Model selection was based on highest validation MLM accuracy across training epochs.

### Position-stratified validation protocol

To mitigate “homology leakage,” we implemented a rigorous position-stratified split for all phenotype precision tasks.

#### Leakage control

Unlike random splits, where mutations at the same site can appear in both training and test sets, position-stratified splitting ensures that all mutations at a specific residue position are held out together.

#### Cross-validation

We utilized **5-fold cross-validation**. Residue positions were randomly partitioned into five subsets, and sequences were assigned to training or testing sets based on whether their mutated positions belonged exclusively to those subsets. To assess sensitivity to fold definition, we repeated the stratified evaluation across three independent random seeds (seeds 0, 1, 2) for fold assignment. Supplementary Fig. 10 confirms that mutation count distributions across partitions remain consistent across seeds and folds, indicating that reported performance metrics are not sensitive to the specific fold construction.

#### Input features

Frozen embeddings from the last hidden layer (or mean embeddings where indicated) served as input to a multi-task FCN to jointly predict binding affinity and expression. The FCN consisted of 5 fully connected layers with hidden dimensions determined by the ESM embedding size (320 for ESM2 8 M, 640 for ESM2 150 M, 1280 for ESM2 650 M, 960 for ESMC 300 M, and 1152 for ESMC 600 M), ReLU activations, and no dropout, trained using mean squared error loss.Within each cross-validation split, the model was reinitialized, trained for 1000 epochs with a learning rate of 1e^-5^, and evaluated on the corresponding test set. Performance metrics, including R^2^ and RMSE, were computed per fold and then averaged across folds.

### Clustering and phylogenetic analysis

#### Latent space visualization

High-dimensional embeddings were projected into 2D space using t-SNE [60] with perplexity optimized between 30 and 750. Clustering accuracies were calculated by identifying the percentage of sequences correctly assigned to their majority clusters (100% for MERS, SARS-CoV-1, SARS-CoV-2, Pangolin Virus, and Hibecovirus; 69.7% for Bat Virus). This yielded an overall clustering accuracy of 73.7% when weighted by the number of sequences in each virus category. We note that t-SNE projections are used here for visualization and exploratory analysis. Clustering was performed in the original high-dimensional embedding space via HDBSCAN, with t-SNE used solely for 2D visualization of the resulting structure.

#### Clustering

HDBSCAN [58] was applied to t-SNE projections to identify de novo clusters. Performance was quantified using the Adjusted Rand Index (ARI) and Silhouette Coefficient (SC). To assess robustness to hyperparameter choices, we performed a systematic grid search over HDBSCAN min_cluster_size and min_sample parameters (Supplementary Figs. 9, 7E–F, 8E–F) and swept t-SNE perplexity from 30 to 750 (Supplementary Figs. 3–5). Lineage separation results were additionally validated across nine independent random downsampling seeds at fixed perplexity (Supplementary Fig. 2). All parameter sweep code is available in the GitHub repository.

#### Phylogenetics

Sequences were aligned using **MUSCLE (v5.3)** with default settings [61]. A maximum likelihood tree was constructed using **IQ-TREE (v2.4.0)** [62] with the **WAG+R2** substitution model recommended by ModelFinder [63] and 1000 ultrafast bootstrap [64].

The tree was midpoint rooted using DendroPy (v5.0.6) [65] and visualized with ggtree (v3.10.1) [66].

### Computational resources and reproducibility

PRIME is implemented in PyTorch Lightning [2.5.1] (https://github.com/Lightning-AI/pytorch-lightning*).* All experiments (excluding UMAP generation and split comparisons post initial embedding of sequences) used Python 3.11.13, PyTorch [2.6.0 + cu124], PyTorch Lightning [2.5.1], and ESM library version [3.2.1]. For UMAP and split comparisons, we used Python 3.13.12, PyTorch [2.11.0 + cu130], and scikit-learn [1.8.0]. UMAP and HDBSCAN GPU versions provided by RAPIDS cuML [26.04.000]. Random seeds for fold assignment were set to 0, 1, and 2 for reproducibility. For model training, random seed was set to 0. Model weights, source code, and curated datasets are available under the MIT license in the GitHub repository (https://github.com/lanl/prime**)** to ensure compliance with FAIR data standards. We used eight NVIDIA A100 GPUs via a Distributed Data Parallel (DDP) strategy implemented in Pytorch Lightning. NVIDIA RTX Pro 6000 GPUs (not using DDP) were used for UMAP generation (available in the repository at https://github.com/lanl/prime/tree/main/notebooks/clustering/blackwell**)** as well as the comparison between random split and position-stratified split (Table 1 and **Supplementary Table 6**). The saved checkpoint with the highest validation accuracy from the ESM-RBD model was used for downstream tasks.

Calculation of binding affinity and expression level errors (Fig. 4C and D) were determined using the formula: (predicted-measured)/measured * 100%.

### LLM statement

LLMs were used to aid code development.

## Supplementary Information

Supplementary Material 1.

Supplementary Material 2.

## Data Availability

All genome sequences and associated metadata in the outbreak dataset are published in GISAID’s EpiCoV database with the GISAID identifier: EPI_SET_240219yp. It is composed of 347,409 individual genome sequences with collection dates ranging from 2019-06-25 to 2023-05-17; data were collected in 202 countries and territories. To view the contributors of each individual sequence with details such as accession number, Virus name, Collection date, Originating Lab and Submitting Lab and the list of Authors, visit doi:10.55876/gis8.240219ypThe reference sequence for SARS-CoV-2 used in this study is hCoV-19/Wuhan/WIV04/2019 (WIV04), the official reference sequence employed by GISAID (EPI_ISL_402124, https://gisaid.org/WIV04).All GISAID sequences and associated metadata used in the BetaCov dataset are published in GISAID’s EpiCoV database with the identifier: EPI_SET_250709on. It is composed of 3 individual genome sequences with collection dates ranging from 2017 to 2019-06-25; the data was collected in 1 countries and territories. To view the contributors of each individual sequence with details such as accession number, Virus name, Collection date, Originating Lab and Submitting Lab and the list of Authors, visit doi: 10.55876/gis8.250709on. PRIME source code is available under the MIT license in the GitHub repository (https://github.com/lanl/prime).
