# Supplementary material for: PRIME: An evaluation framework for protein representation inference and generalization in viral mutation space
Source: BMC Genomics. 2026 May 30;27:645. doi: 10.1186/s12864-026-12976-5 (PMC13425921; doi:10.1186/s12864-026-12976-5)
Supplement: Supplementary file 1 — Supplementary Material 1. [file 12864_2026_12976_MOESM1_ESM.docx]

**Supplementary Figures**


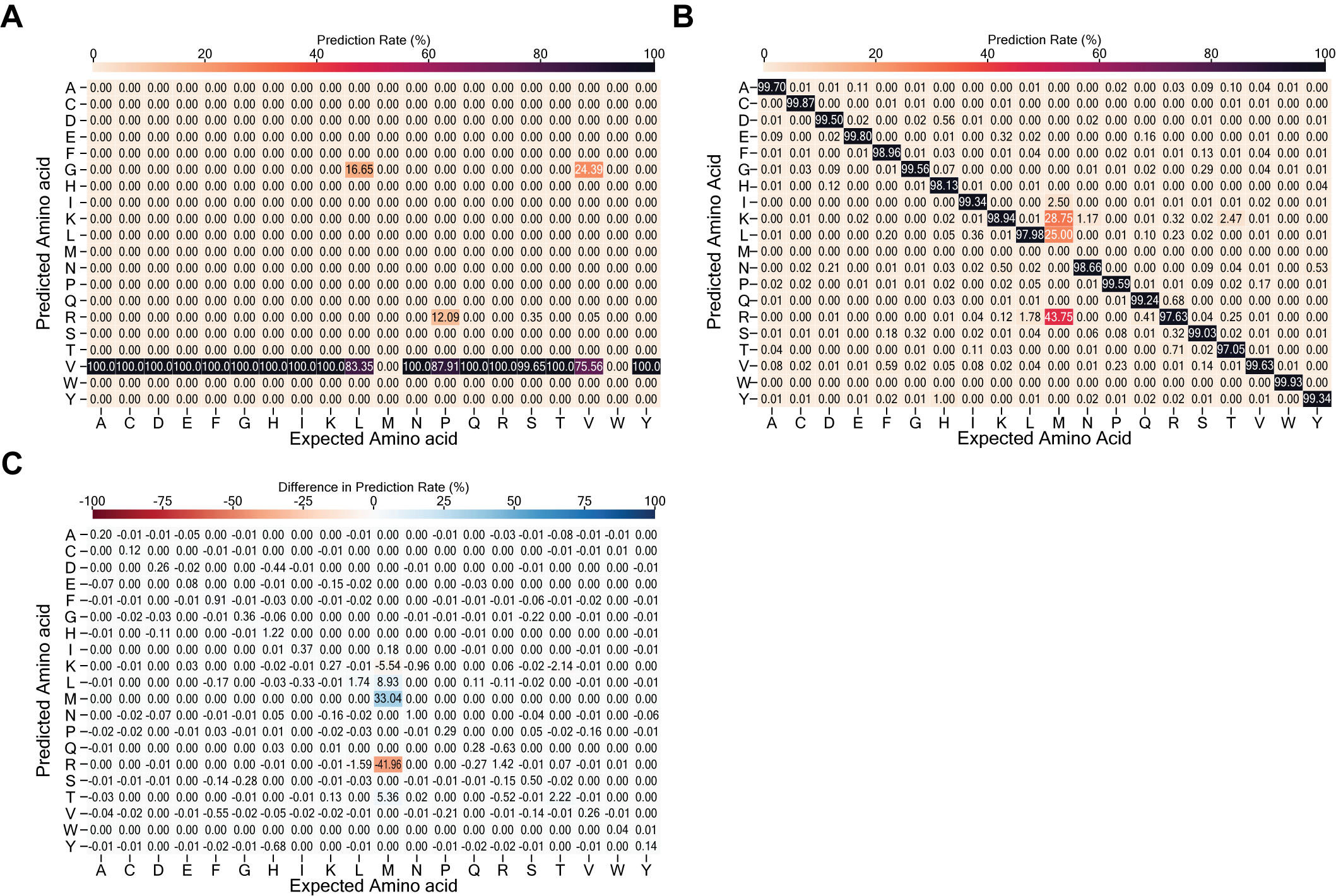


**Supplementary Figure 1. Confusion matrices for masked language BERT model on the RBD dataset. A**. Confusion matrix of the BERT-RBD model MLM accuracy before training, with overall accuracy of 3.78% from the correct predictions from diagonal values. **B.** Confusion matrix of the fine-tuned BERT-RBD MLM accuracy reached 94.09% after 100 epochs of training. **C.** Difference in prediction rate for confusion matrices for the fine-tuned BERT-RBD model and the fine-tuned ESM-RBD model on the RBD dataset (ESM-RBD prediction rates minus BERT-RBD prediction rates).

**
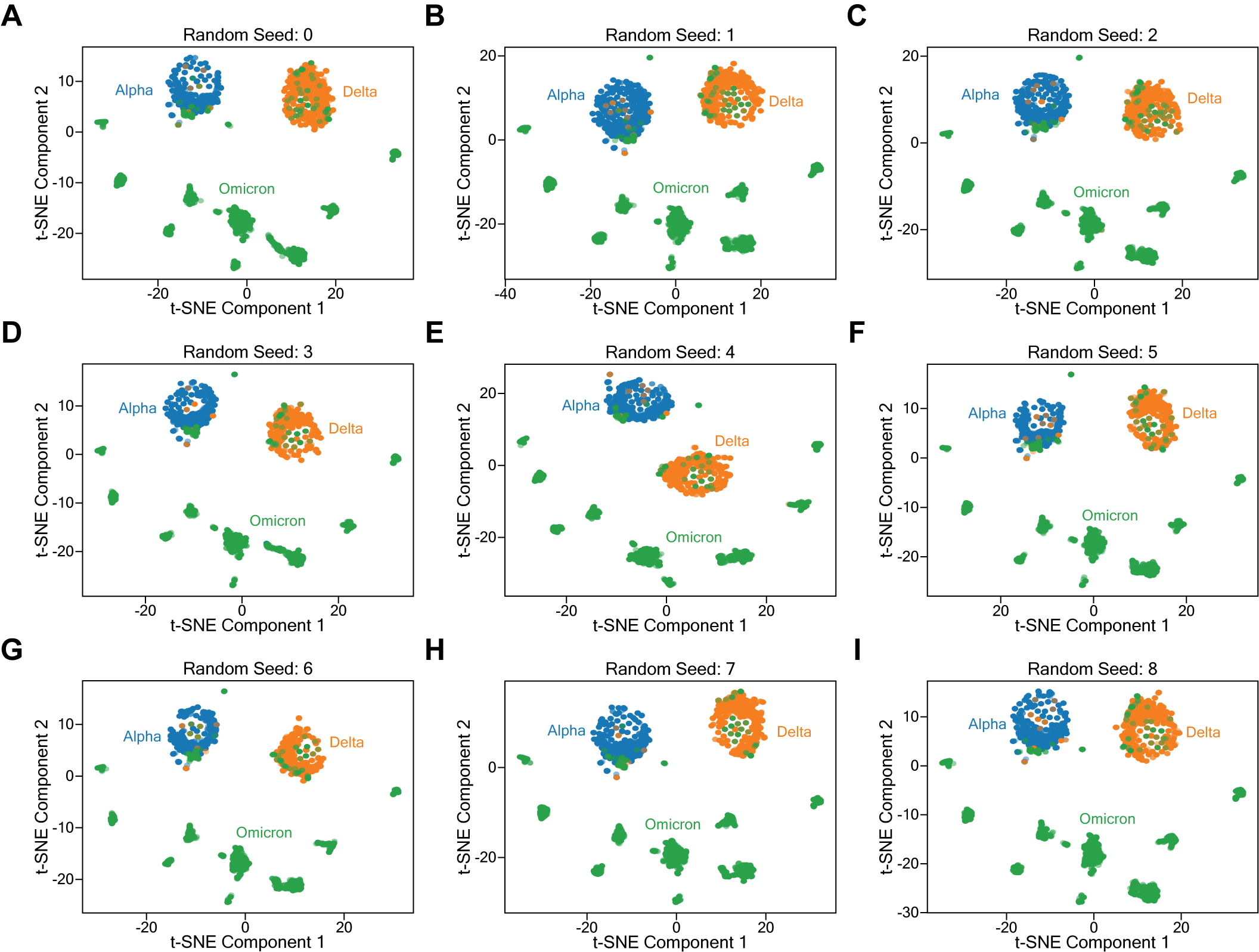
Supplementary Figure 2. t-SNE visualizations of SARS-CoV-2 RBD variants (Alpha, Delta, Omicron) at a fixed perplexity of 750 across different random data samples.** t-SNE visualization of sequence-level latent representations derived from the last hidden state of the ESM model. Three major SARS-CoV-2 lineages were balanced through random downsampling (n = 22,075 sequences per lineage). Panels **A-I** each show a different random subset (random seed value indicated in panel titles) of the RBD sequences, and illustrate the effect on the visual separation between Alpha (blue), Delta (orange), and Omicron (green) lineages. Across all of the panels, the three clusters are consistently well separated at the fixed perplexity of 750, regardless of the random sampling.

**
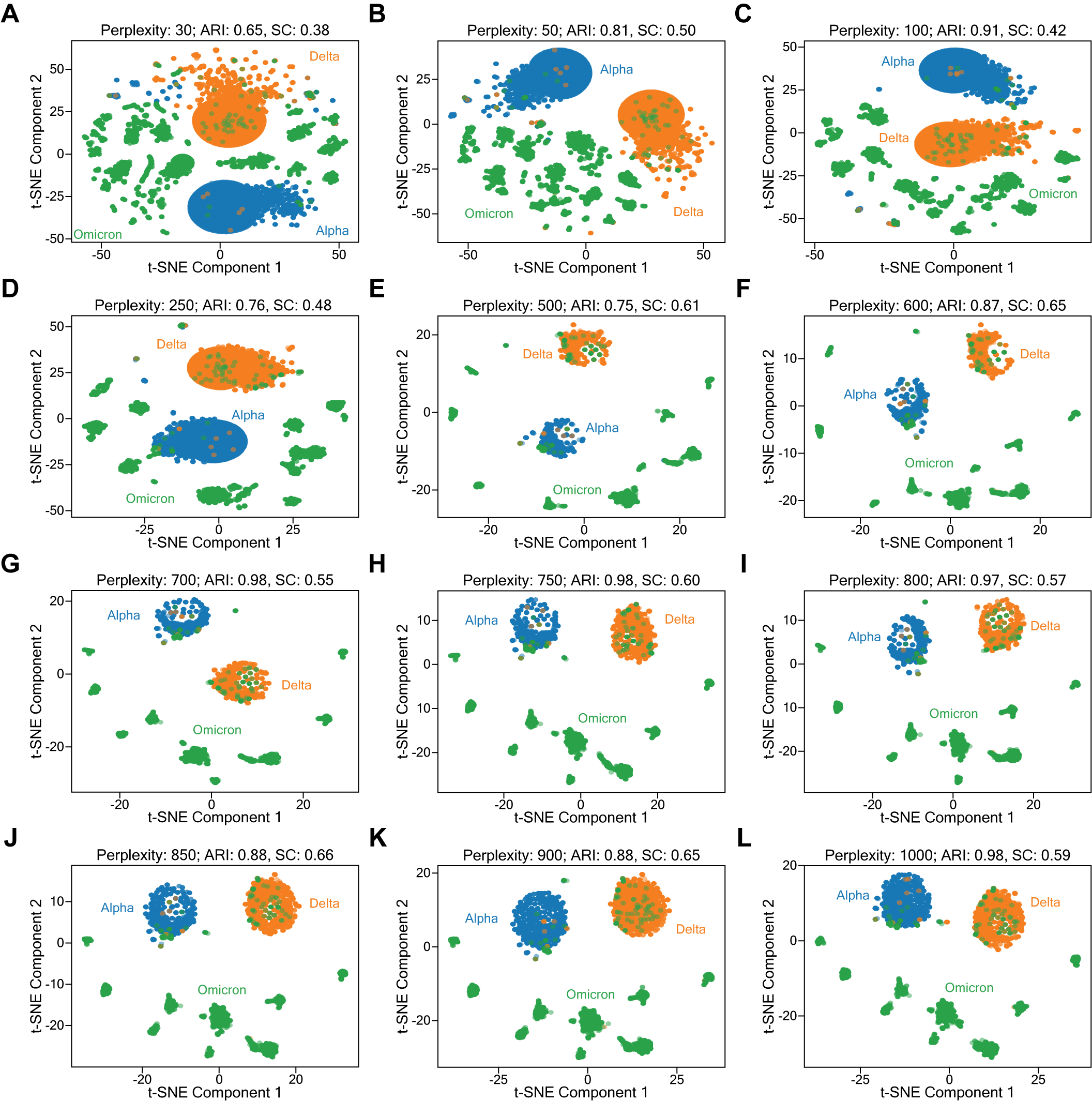
**

**Supplementary Figure 3. Effect of t-SNE perplexity on clustering of SARS-CoV-2 RBD variants (Alpha, Delta, Omicron) using ESM-RBD embeddings.** t-SNE visualization of sequence-level latent representations derived from the last hidden state of the ESM model. Three major SARS-CoV-2 lineages were balanced through random downsampling (n = 22,075 sequences per lineage). Each panel shows a different perplexity value and its effect on the visual separation between Alpha (blue), Delta (orange), and Omicron (green) lineages. The best HDBSCAN ARI and corresponding SC scores were also calculated for each perplexity. Several perplexities had the same ARI score; in this case, SC scores helped to determine the best plot. **A-F**. Perplexity and corresponding ARI and SC scores shown in each panel title.

**
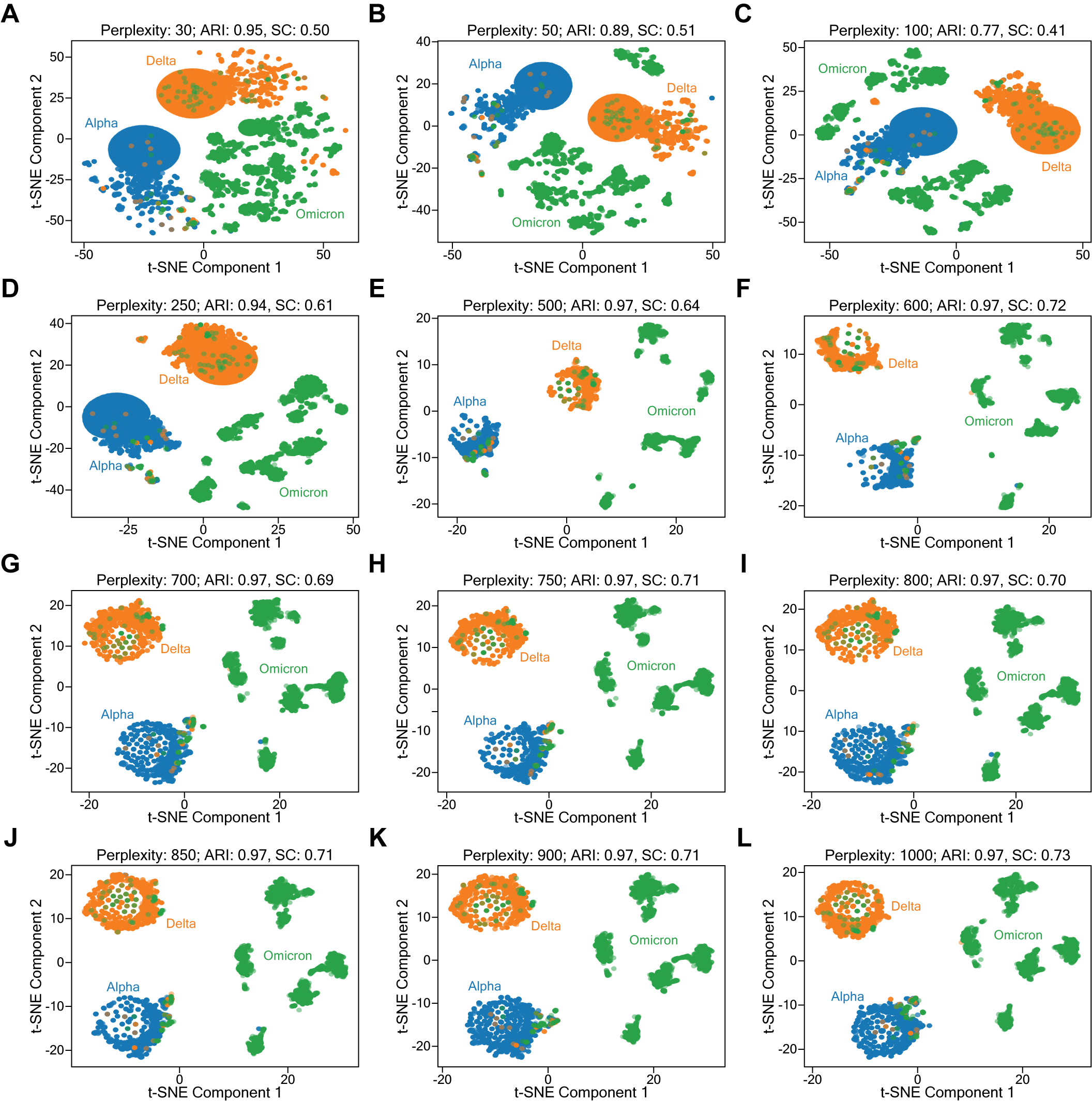
Supplementary Figure 4. Effect of t-SNE perplexity on clustering of SARS-CoV-2 RBD variants (Alpha, Delta, Omicron) using non-fine-tuned ESM embeddings.** t-SNE visualization of sequence-level latent representations derived from the last hidden state of the ESM model. Three major SARS-CoV-2 lineages were balanced through random downsampling (n = 22,075 sequences per lineage). Each panel shows a different perplexity value and its effect on the visual separation between Alpha (blue), Delta (orange), and Omicron (green) lineages. The best HDBSCAN ARI and corresponding SC scores were also calculated for each perplexity. Several perplexities had the same ARI score; in this case, SC scores helped to determine the best plot. **A-F.** Perplexity and corresponding ARI and SC scores shown in each panel title.

**
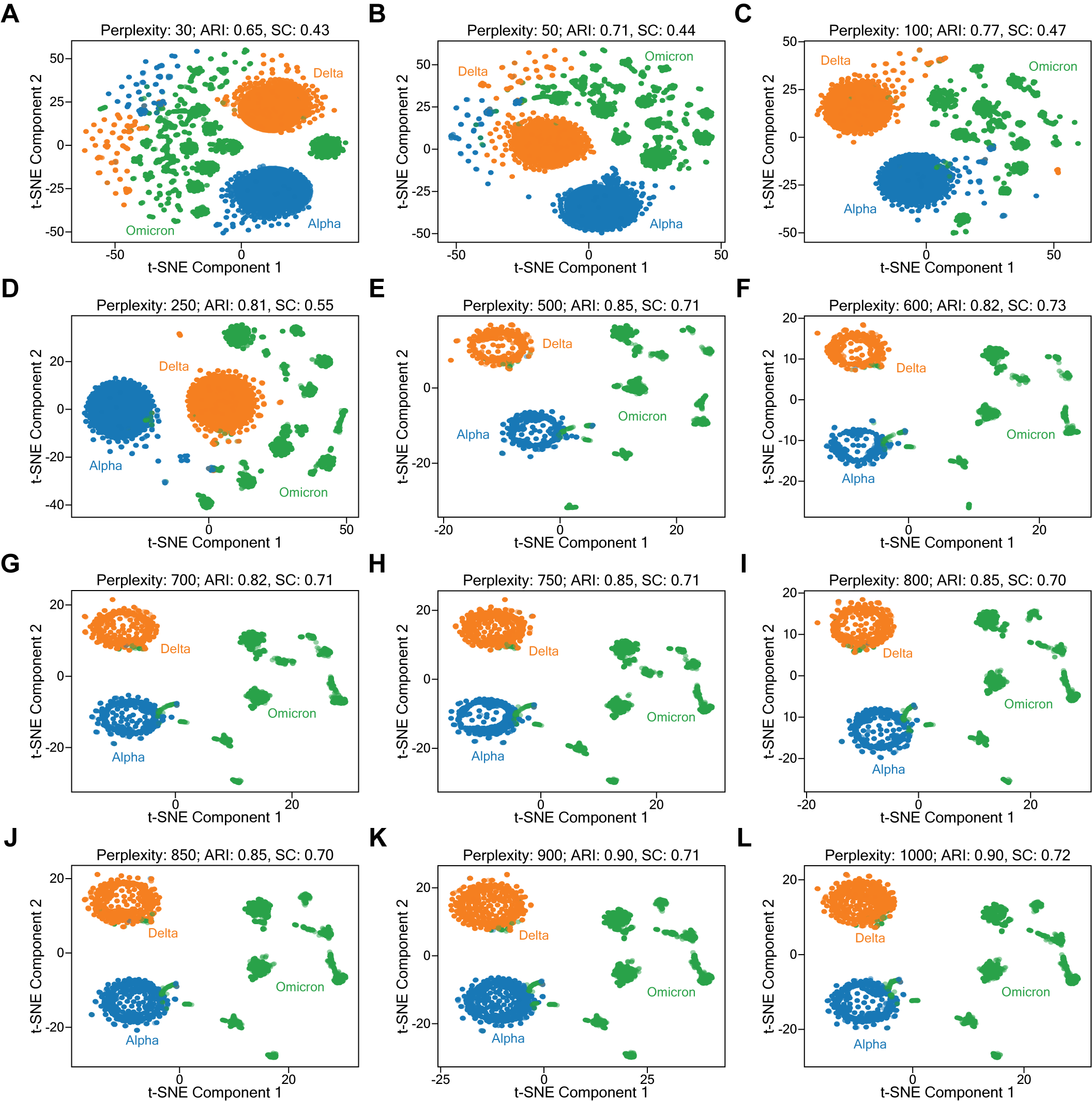
Supplementary Figure 5. Effect of t-SNE perplexity on clustering of SARS-CoV-2 RBD variants (Alpha, Delta, Omicron) using MSA with One-Hot.** t-SNE visualization of sequence-level latent representations derived from one-hot encoding of the MSA. Three major SARS-CoV-2 lineages were balanced through random downsampling (n = 22,075 sequences per lineage). Each panel shows a different perplexity value and its effect on the visual separation between Alpha (blue), Delta (orange), and Omicron (green) lineages. The best HDBSCAN ARI and corresponding SC scores were also calculated for each perplexity. Several perplexities had the same ARI score; in this case, SC scores helped to determine the best plot. The HDBSCAN t-SNE plots are not shown here. **A-F**. Perplexity and corresponding ARI and SC scores shown in each panel title.


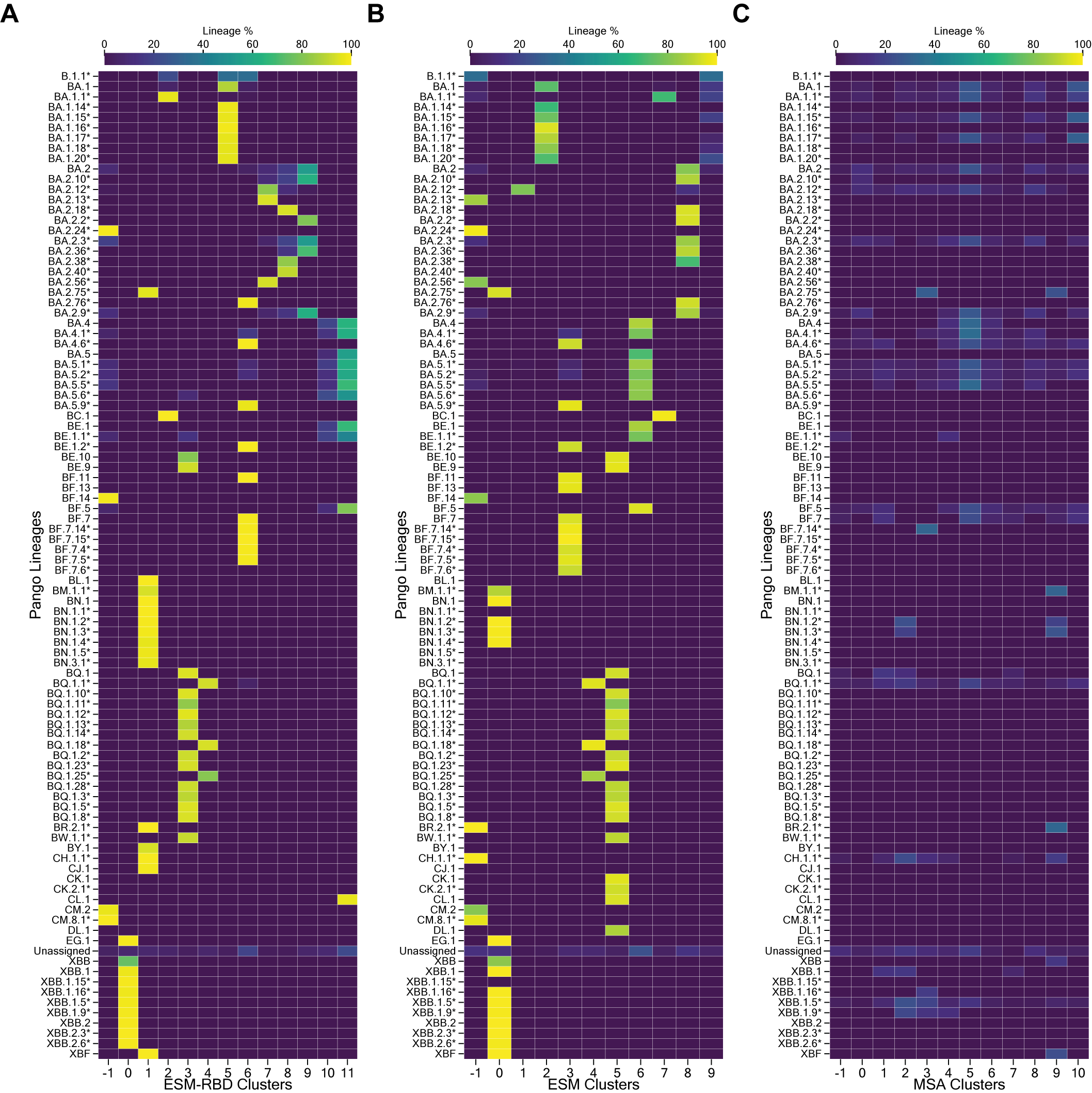


**Supplementary Figure 6. Heatmaps comparing lineage % of SARS-CoV-2 Omicron subclusters across ESM-RBD, non-fine-tuned ESM, and MSA with One-Hot methods.** Corresponds to Supplementary Table 2. Lineage % defined as the number of sequences of a specific Pango lineage within a given cluster divided by the total number of sequences of that Pango lineage across all clusters, multiplied by 100. Empty/NaN values were filled in as 0%. **A.** ESM-RBD clusters. **B.** non-fine-tuned ESM clusters. **C.** MSA with One-Hot clusters.


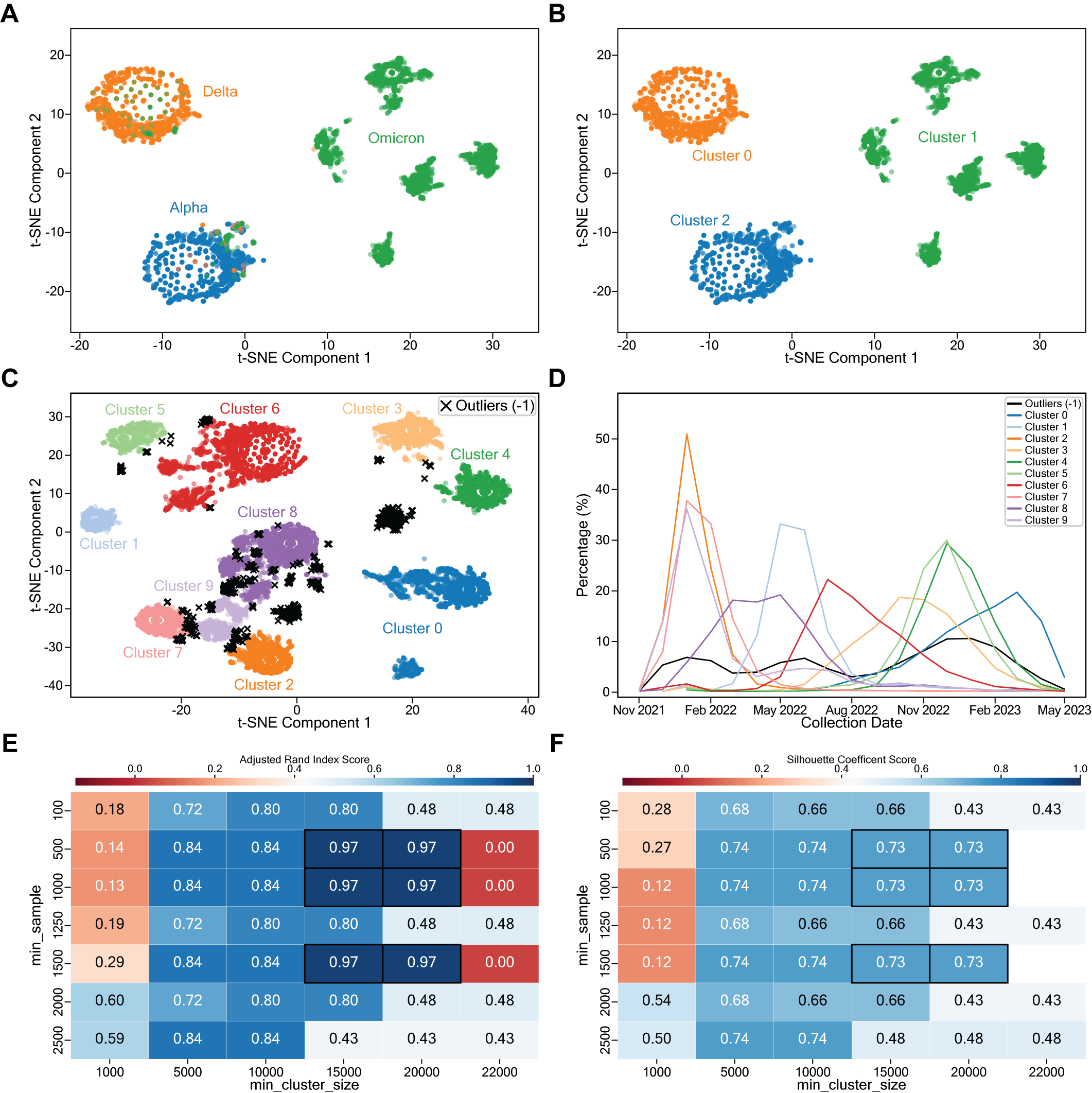


**Supplementary Figure 7.** **Clustering of SARS-CoV-2 RBD sequences from the non-fine-tuned ESM model. A.** t-SNE visualization of sequence-level latent representations derived from the last hidden state of the non-fine-tuned ESM model. Alpha (blue), Delta (orange), and Omicron (green) lineages are visually separated. **B.** HDBSCAN clustering applied to the t-SNE representations identifies three primary clusters corresponding to the major lineages, without outliers (black X). Cluster 0 (orange) corresponds predominantly to Delta, cluster 1 (green) to Omicron, and cluster 2 (blue) to Alpha. **C.** HDBSCAN clustering applied to t-SNE representations of all available Omicron sequences (n = 160,016) reveals 10 distinct clusters (0-9) and outliers. **D.** Temporal dynamics of Omicron clusters from November 2021 to May 2023, showing the percentage of sequences within each cluster over time. **E.** and **F.** ARI and SC parameter scan.

**
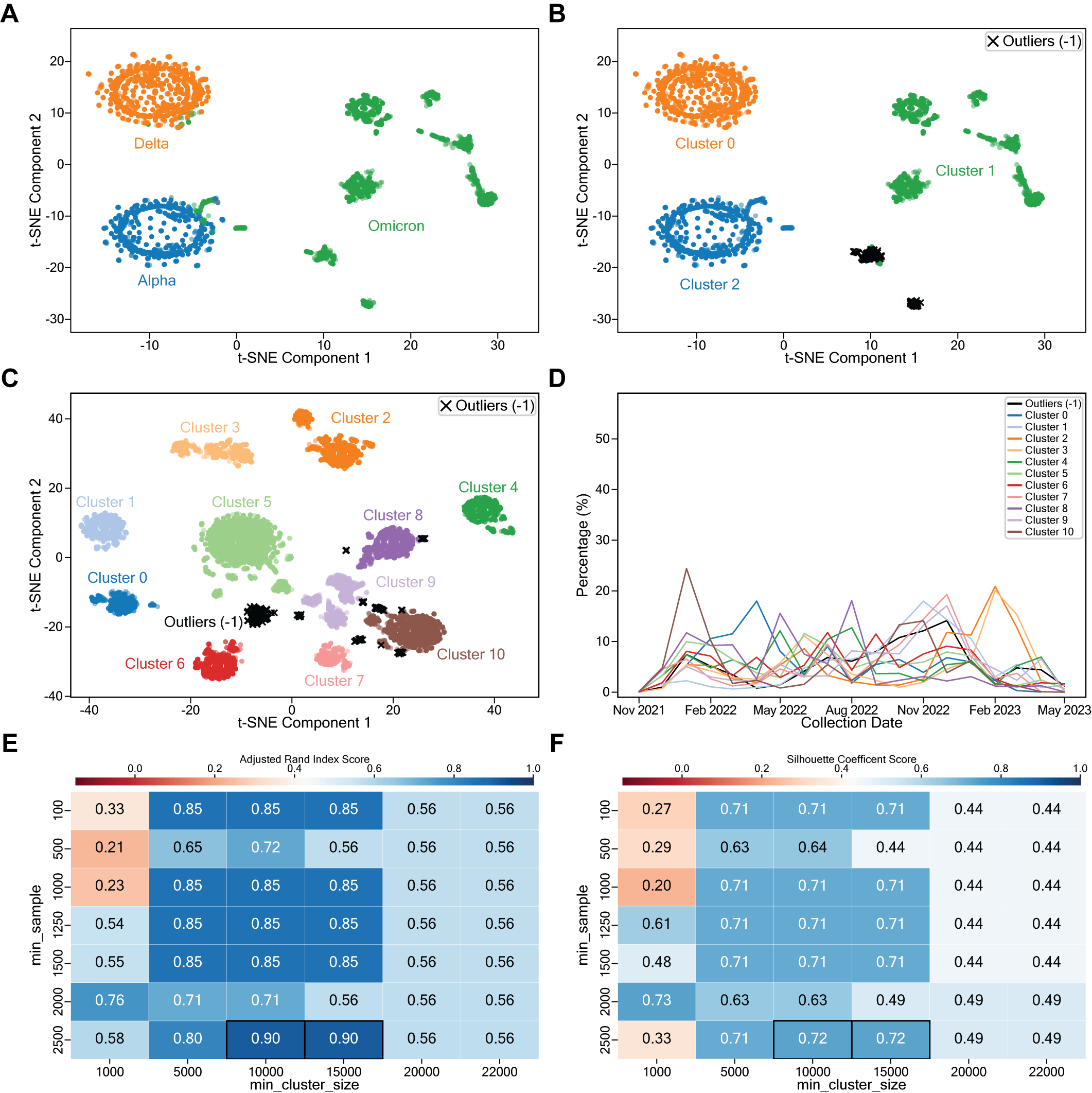
**

**Supplementary Figure 8.** **Clustering of SARS-CoV-2 RBD sequences using MSA and One-Hot Encoding. A.** t-SNE visualization of sequence-level latent representations derived from one-hot encoding of the MSA. Alpha (blue), Delta (orange), and Omicron (green) lineages are visually separated. **B.** HDBSCAN clustering applied to the t-SNE representations identifies three primary clusters corresponding to the major lineages, with a small number of outliers (black X). Cluster 0 (orange) corresponds predominantly to Delta, cluster 1 (green) to Omicron, and cluster 2 (blue) to Alpha. **C.** HDBSCAN clustering of all available Omicron sequences (n = 160,016) reveals 11 distinct clusters (0-10) and outliers. **D.** Temporal dynamics of Omicron clusters from November 2021 to May 2023, showing the percentage of sequences within each cluster over time. The succession of dominant clusters reflects the evolutionary trajectory of the Omicron lineage. **E.** and **F.** ARI and SC parameter scan.


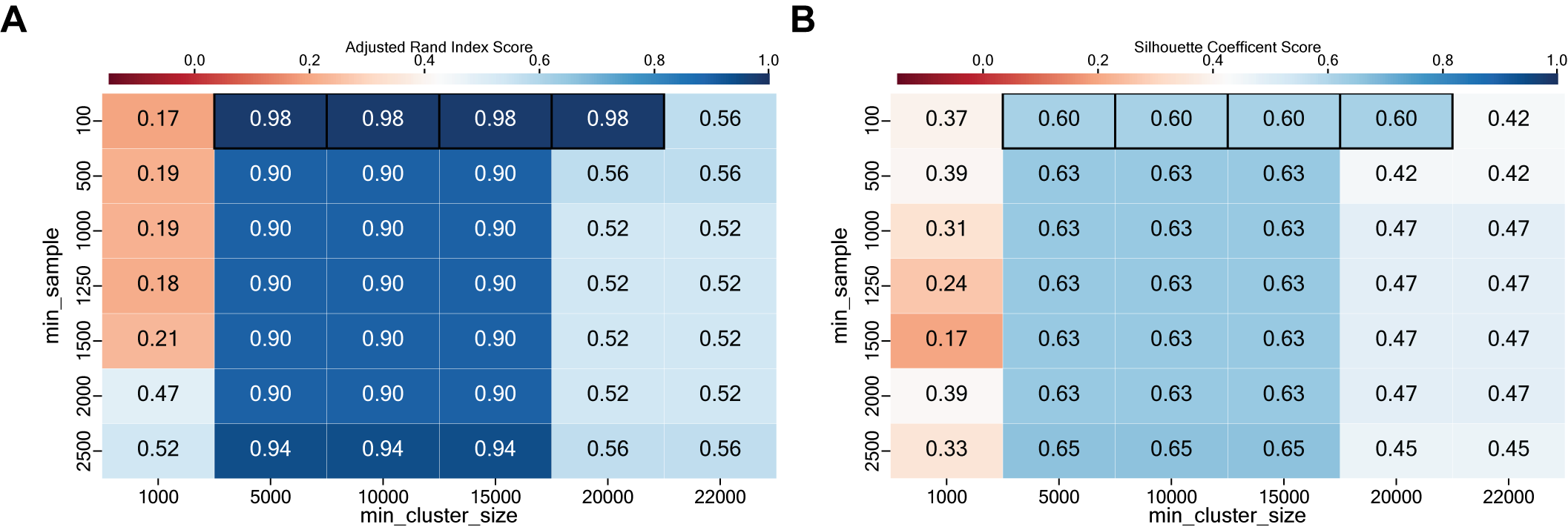


**Supplementary Figure 9. Determination of HDBSCAN ARI and corresponding SC scores for the fine-tuned ESM-RBD clustering of the SARS-CoV-2 RBD sequences in Fig. 2A and B. A.** Adjusted Rand Index heatmap for determining the best combination of HDBSCAN parameters min_cluster_size and min_sample. Black highlighted region is the best determined ARI (0.98). **B.** Silhouette Coefficient heatmap for determining the best combination of HDBSCAN parameters min_cluster_size and min_sample. Black highlighted region is the best SC (0.60) based on the best determined ARI from **A.**

**
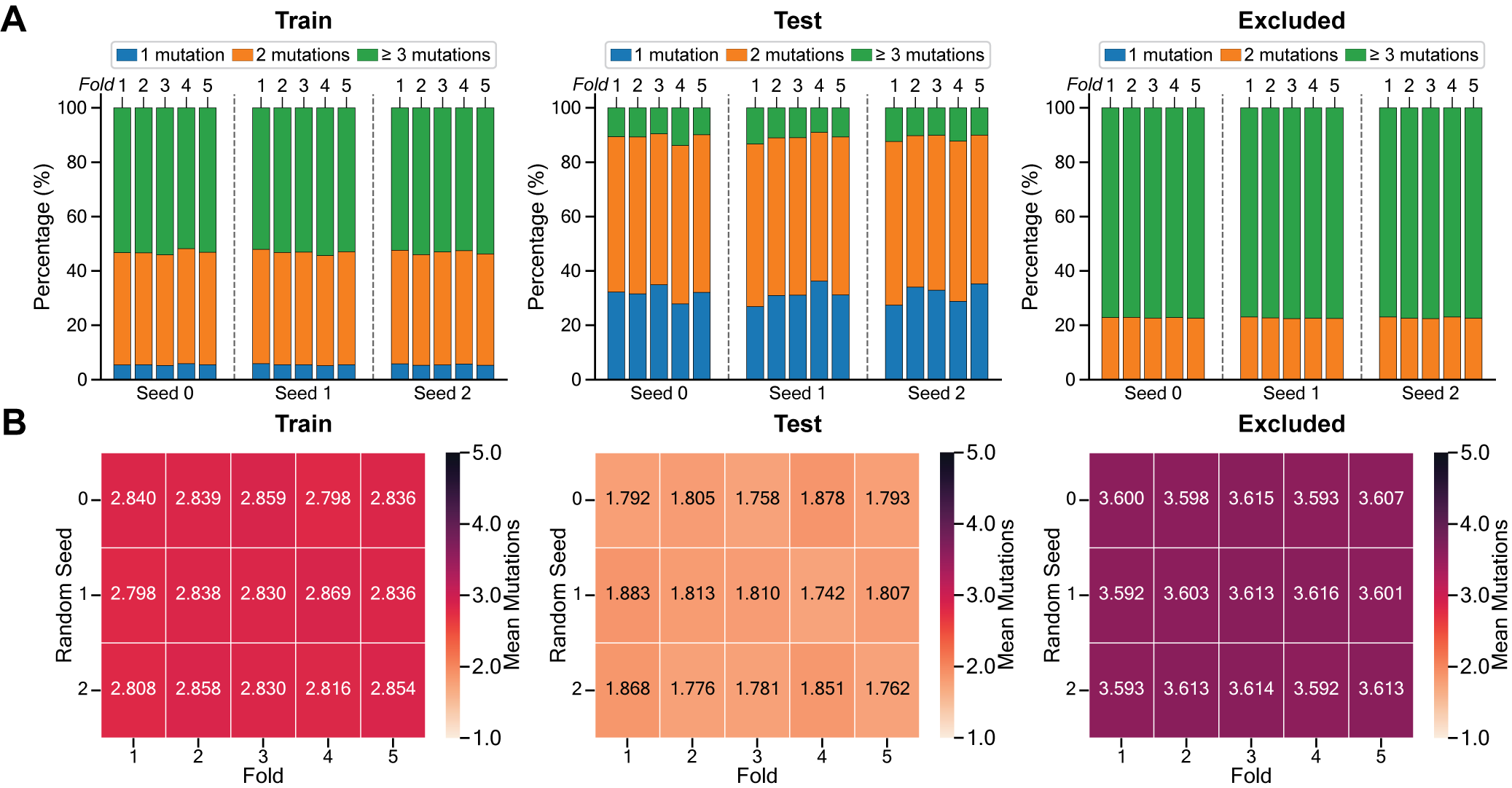
**

**Supplementary Figure 10. Mutation distribution and consistency across random seeds and folds. A.** Distribution of mutation counts (1, 2, ≥3 mutations) across folds (1-5) for each random seed (0-2) within Train, Test and Excluded partitions. Stacked bar plots show the percentage composition of mutation categories per fold. **B.** Mean number of mutations per random seed-fold combination for Train, Test, and Excluded partitions. Heatmaps indicate a consistent number of mutations across folds and seeds within each partition.
